# Micronutrient status in children aged 6–59 months with severe wasting and/or nutritional edema: implications for nutritional rehabilitation formulations

**DOI:** 10.1093/nutrit/nuad165

**Published:** 2024-02-13

**Authors:** Laura Vresk, Mary Flanagan, Allison I Daniel, Isabel Potani, Celine Bourdon, Carolyn Spiegel-Feld, Mehakpreet K Thind, Amber Farooqui, Catriona Ling, Emiliano Miraglia, Guanlan Hu, Bijun Wen, Stanley Zlotkin, Philip James, Marie McGrath, Robert H J Bandsma

**Affiliations:** Translational Medicine, The Hospital for Sick Children, Toronto, Ontario, Canada; Translational Medicine, The Hospital for Sick Children, Toronto, Ontario, Canada; Translational Medicine, The Hospital for Sick Children, Toronto, Ontario, Canada; Department of Nutritional Sciences, Temerty Faculty of Medicine, University of Toronto, Toronto, Ontario, Canada; Translational Medicine, The Hospital for Sick Children, Toronto, Ontario, Canada; Translational Medicine, The Hospital for Sick Children, Toronto, Ontario, Canada; Translational Medicine, The Hospital for Sick Children, Toronto, Ontario, Canada; Translational Medicine, The Hospital for Sick Children, Toronto, Ontario, Canada; Translational Medicine, The Hospital for Sick Children, Toronto, Ontario, Canada; Translational Medicine, The Hospital for Sick Children, Toronto, Ontario, Canada; Translational Medicine, The Hospital for Sick Children, Toronto, Ontario, Canada; Translational Medicine, The Hospital for Sick Children, Toronto, Ontario, Canada; Translational Medicine, The Hospital for Sick Children, Toronto, Ontario, Canada; Department of Nutritional Sciences, Temerty Faculty of Medicine, University of Toronto, Toronto, Ontario, Canada; Emergency Nutrition Network, Oxford, United Kingdom; Emergency Nutrition Network, Oxford, United Kingdom; Translational Medicine, The Hospital for Sick Children, Toronto, Ontario, Canada; Department of Nutritional Sciences, Temerty Faculty of Medicine, University of Toronto, Toronto, Ontario, Canada

**Keywords:** Severe malnutrition, severe acute malnutrition, severe wasting, nutritional edema, kwashiorkor, marasmus, pediatric, therapeutic feeds, micronutrient status, micronutrient deficiency, micronutrient toxicity, sodium, potassium, calcium, phosphorus, magnesium, iron, copper, zinc, selenium, iodine, thiamine, riboflavin, niacin, pantothenic acid, vitamin B_6_, folic acid, vitamin B_12_, biotin

## Abstract

Undernutrition remains a global struggle and is associated with almost 45% of deaths in children younger than 5 years. Despite advances in management of severe wasting (though less so for nutritional edema), full and sustained recovery remains elusive. Children with severe wasting and/or nutritional edema (also commonly referred to as severe acute malnutrition and part of the umbrella term “severe malnutrition”) continue to have a high mortality rate. This suggests a likely multifactorial etiology that may include micronutrient deficiency. Micronutrients are currently provided in therapeutic foods at levels based on expert opinion, with few supportive studies of high quality having been conducted. This narrative review looks at the knowledge base on micronutrient deficiencies in children aged 6–59 months who have severe wasting and/or nutritional edema, in addition to highlighting areas where further research is warranted (See “Future Directions” section).

## INTRODUCTION

Undernutrition remains an important global health burden. It contributes directly or indirectly to 45% of all mortality in children under the age of 5 years.^1^ Severe malnutrition is defined broadly as any form of undernutrition associated with a high risk of severe adverse outcomes.^2^ Severe malnutrition is an umbrella term, which, along with stunting (ie, low height for age), underweight (ie, low weight for age) and micronutrient forms of undernutrition, also includes wasting (ie, low weight for height; also known as marasmus), small mid-upper arm circumference, and nutritional edema (also known as kwashiorkor) manifestations.^2^ The term *severe acute malnutrition* (SAM) is defined in children aged 6–59 months as a weight-for-height *z* score of less than –3, a mid-upper arm circumference of less than 115mm, and/or the presence of bilateral pitting edema.^3^ SAM in infants younger than 6 months has different defining parameters, different treatment options, and likely different underlying etiology and pathophysiology^4^; SAM in infants is not included in this review. For the purposes of this review, we use the terms *severe malnutrition*, *severe wasting* and/or *nutritional edema*, and SAM interchangeably. Here, we do not discuss the other forms or manifestations of severe malnutrition.

In 2023, the global joint UNICEF, World Health Organization (WHO), World Bank Group estimate of children under the age of 5 years who had severe wasting was 13.7 million.^5^ A large cross-sectional study of 94 low- and middle-income countries (LMICs) that included 804 172 children showed a 14% prevalence of wasting in children younger than 2 years, compared with 9% in children aged 2–4 years.^6^ There are no accurate data on the global prevalence of nutritional edema.^7^ Risk of death is exceptionally high, at 10%–23%, in severely malnourished children during hospitalization^8–11^ but also remains high after discharge.^9^^,^^11^ Surviving children also experience lasting consequences such as impaired linear growth, functional deficits, and poor developmental outcomes.^11–13^

Medically stable children with SAM (also referred to as “uncomplicated” SAM) can be treated in outpatient/community settings with ready-to-use therapeutic food (RUTF). Sick children with SAM (or “complicated” SAM), defined as severe wasting and/or nutritional edema with appetite loss, severe edema, or the presence of medical complications, require inpatient treatment.^14^^,^^15^ Treatment first involves stabilization and management of urgent medical conditions. During this stabilization phase, children are fed therapeutic milk F-75 orally or enterally (130 mL/kg/d = ∼100 kcal/kg/d).^15^^,^^16^ Following stabilization, children are gradually transitioned to high-calorie therapeutic foods, either F-100 and/or RUTF, for their rehabilitation phase (∼135–220 kcal/kg/d).^15^ Once children are well established with intake of their rehabilitation phase foods, they are discharged home and continue rehabilitation with RUTF.^14^^,^^16^

Micronutrients are essential for health; deficiencies in various micronutrients can have acute and chronic effects on different organ systems in the body.^17^ Micro- and macronutrient deficiencies often coexist in severe malnutrition and their effects on morbidity and mortality may be intertwined or compounded. Unfortunately, there are limited high-quality data on the prevalence and degree of severity of micronutrient deficiencies in severely malnourished children.

F-75, F-100, and RUTF all contain micronutrients to potentially prevent or treat deficiencies, reduce the risk of refeeding syndrome, and promote homeostasis.^14^^,^^15^^,^^17^ The dose of each micronutrient in these therapeutic foods is based largely on expert opinion rather than on direct evidence. The most recent 2013 WHO recommendations are that supplementation outside of therapeutic feeding can be considered depending on clinical signs and symptoms of overt deficiency.^15^

Since their development in the 1990s, there has never been a comprehensive examination of all micronutrients in F-75, F-100, and RUTF, to our knowledge, in relation to requirements for children with SAM throughout the treatment pathway. Although anthropometric recovery is often a metric of successful treatment of SAM, nutritional recovery more broadly including micronutrient status should also be considered. Therefore, determining the appropriate quantity of micronutrients in therapeutic foods is fundamental to understanding how to promote complete nutritional recovery in children with SAM. We assessed the evidence on deficiencies and requirements of specific micronutrients in children with SAM and how the amounts of specific micronutrients added to nutritional rehabilitation formulations compare with their micronutritional needs.

## METHODS

To provide a practical understanding of intake of specific micronutrients in children with SAM, we used information published from Nutriset’s website^18^ to determine the micronutrient content of standard preparations of F-75, F-100, and RUTF currently formulated (see Table S1 in the Supporting Information online), and we compared intake to the Dietary Reference Intakes (DRIs). Of note, Nutriset is 1 of more than 20 commercial manufacturers of RUTF,^19–21^ and its RUTF product is commonly used worldwide. Recently, new Codex specifications for RUTF have been published, laying out both minimum and maximum micronutrient constituents of RUTF.^22^ Nutriset’s RUTF contains micronutrients that lie within these Codex-specified ranges (see Table S2 in the Supporting Information online). However, it is important to note that Nutriset quotes their stated volumes of constituents are “based on Nutriset’s knowledge of the intrinsic nutrient content of the raw materials and their variability, as well as the variability of the process.”^18^ Of note, no Codex specifications exist for F-75 and F-100 formulations.

The DRIs are a set of nutrient-based reference values that can be used to provide guidelines for nutrient intake across the lifespan and are based on both sex and age of healthy individuals. Specifically, the DRIs include the estimated average requirement, the Recommended Daily Allowance (RDA), the Adequate Intake (AI), and the upper limit (UL).^23^^,^^24^ Descriptions of each DRI can be found in Table S3 in the Supporting Information online. Although there are limitations to using the DRIs, most notably that they are based on healthy rather than sick populations,^24^ they were chosen as a universal benchmark to compare intake to recommendations to establish some consistent comparisons. This is in keeping with best practice.^25^

**Figure 1:**
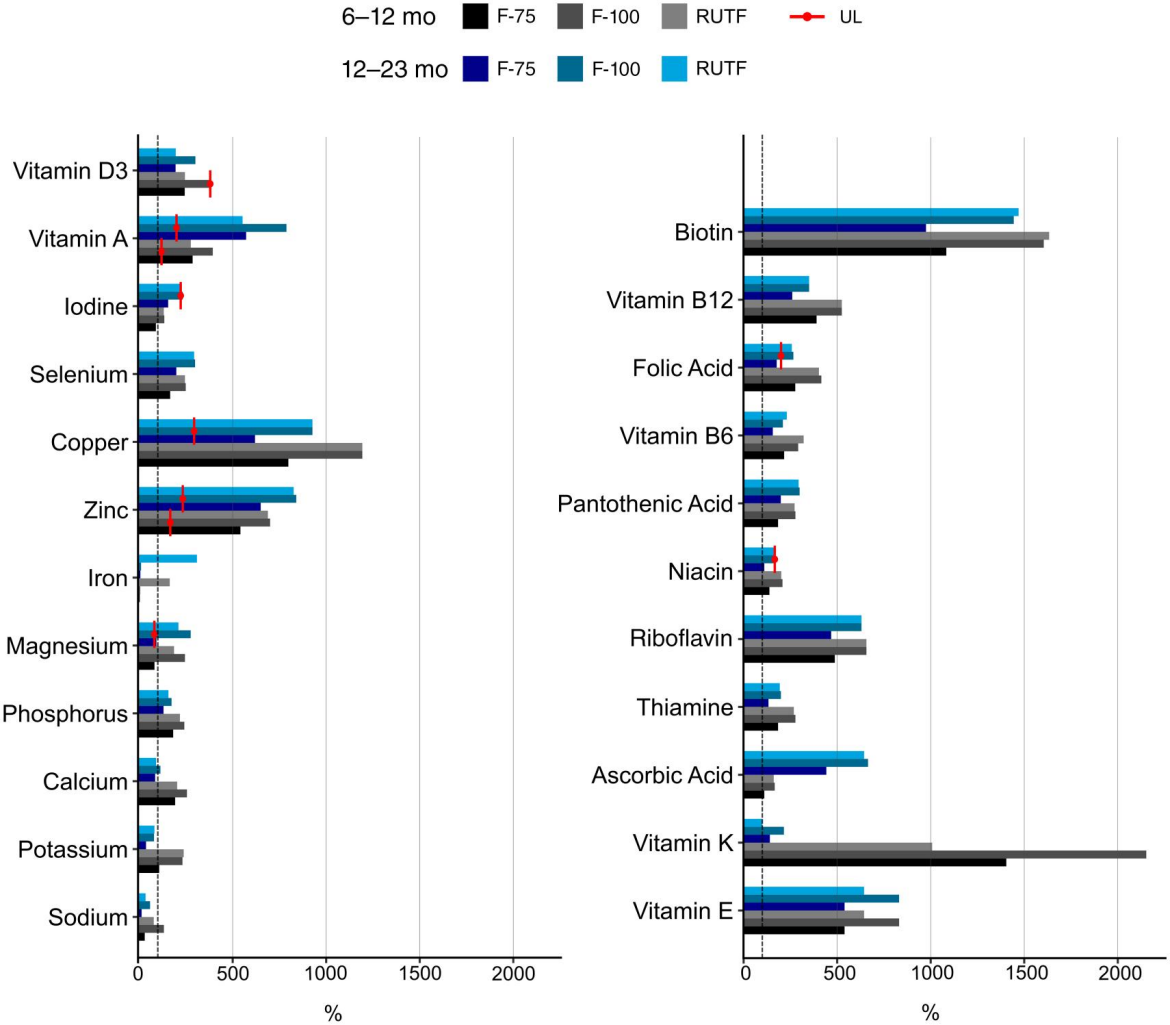
**Estimated micronutrient intake as percentage of the Recommended Daily Allowance or Adequate Intake provided for each food type and for age groups 6–12 and 12–23 months.** *Abbreviation:* RUTF, ready-to-eat food.

For some micronutrients, a range of content in Nutriset’s therapeutic products F-75 and F-100 is provided due to variability in raw materials and processing, and so the midpoint of these ranges was used for calculations of intake. We then used realistic weights of children based on data from a large international cohort study including infants and children, aged 6–23 months, with severe wasting in Africa and South Asia,^9^ and from a subset of data on children 24–59 months old from a study in Malawi^26^ to determine average prescriptions for daily intakes of F-75, F-100, and RUTF (Table 1). This allowed us to estimate a realistic daily amount of each micronutrient that a child with SAM would consume across the different phases of treatment and compare it with the RDA or AI and UL of each micronutrient (Figure 1, see Tables S4–S12 in the Supporting Information online).^27–32^ Limited robust methodology data exist on the volumes of therapeutic foods consumed vs the volumes prescribed, though it is generally believed that children infrequently consume the prescribed volumes. One study of 780 children admitted to hospital with SAM reported that 58% children were not able to complete prescribed feedings, with feeding completion defined as consumption of at least 75% prescribed volumes.^33^ Therefore, the tables in this review possibly overestimate the percentages of RDAs or AIs consumed for each micronutrient. Additionally, the percentages of RDA or AIs for children with SAM aged 12–23 months were compared with the DRIs developed for healthy children aged 1–3 years. Given the median weight of 6.1 kg is more indicative of an infant younger than 12 months, the percentages of RDA or AI intake may be underestimated. A weight of 8.5 kg was used for volume calculations of children aged 24–59 months.^26^ The DRIs for this age group were compared with the DRIs for healthy children aged 1–3 years, because 70% of the children 24 months old or older included in the database were younger than 36 months.^26^ For this reason, the tables provided may overestimate the percentage of RDA or AI micronutrient intake for children aged 36–59 months.

**Table 1:** Amount and type of therapeutic food prescribed per SAM treatment phase

Age (mo)	Weight (kg)	Phase	Stabilization	Rehabilitation
		Food type	F-75 (75 kcal/100 mL)	F-100 (100 kcal/100 mL)^a^ or RUTF (500 kcal/92 g sachet)
		Volume or kcal prescribed per day	130 mL/kg/d (∼100 kcal/kg/d)^15^	135-220 kcal/kg^a^ 175 kcal/kg/d^b^
6–12	∼5 kg^9^	Estimated intake per day	650 mL	875 mL F-100 or 1.75 sachets RUTF
12–23	∼6 kg^9^		780 mL	1050 mL F-100 or 2.1 sachets RUTF
23–59	∼8.5 kg^26^		1105 mL	1500 mL F-100 or 3 sachets RUTF

a F-100 is used in rehabilitation phase for few patients. Note the transition phase (F-100 or RUTF) is not included, because a relatively short duration generally was observed.

b Midpoint of prescribed amount.

*Abbreviations:* RUTF, ready-to-eat therapeutic food; SAM, severe acute malnutrition.

We reviewed the literature by searching the MEDLINE database with the following terms: (“protein-energy malnutrition” or “severe acute malnutrition” or “severe malnutrition” or “kwashiorkor” or “marasmus” or “severe wasting” or “nutritional edema”) AND different variations (“*micronutrient X*”) of each micronutrient to address questions about 4 key elements of micronutrients and SAM. In addition, the reference list of each included article was evaluated to determine if articles were missed. Articles met inclusion criteria if the study reported on in the article included assessment of micronutrient intake, micronutrient status, or response to nutritional rehabilitation by children with SAM. Articles were excluded if they were not published in the English language or if they were focused on adult populations.

For each micronutrient, we reviewed the following 4 elements: (1) overview of micronutrient: what is the physiological function of micronutrient X in children? How is deficiency or excess of micronutrient X determined? (2) Evidence on the micronutrient status in SAM: what is the evidence on deficiencies and requirements of micronutrient X in children with SAM? (3) Comparison to recommendations: how does the predicted daily intake of micronutrient X in F-75, F-100, and RUTF compare with the DRIs? What are the implications of these levels of intake? And (4) interpretation and discussion: is the content of micronutrient X in F-75, F-100, and RUTF appropriate? Based on the evidence, are there recommendations for further research or revision of formulas or supplementation?

## RESULTS

### Sodium

Hyponatremia and hypernatremia are generally defined as a serum sodium concentration of less than 135 mmol/L or greater than 150 mmol/L, respectively, with slightly varying ranges depending on the reporting laboratory.^34^ Rapid, large changes in sodium levels can lead to cerebral edema characterized by nausea, confusion, seizures, and coma, whereas gradual changes in sodium levels are better tolerated but can lead to vomiting, fatigue, confusion, and headaches.^34^

Children with SAM are thought to have excess body sodium caused by high intracellular sodium; this perhaps is more pronounced in children with nutritional edema, but supporting evidence is limited.^35^^,^^36^ It has been postulated to occur through different adaptive processes. In severe wasting, inhibition and/or slowing of the sodium pump has been reported. In nutritional edema, although an increase in the activity of the Na^+^/K^+^-ATPase has been found, leakiness of the cellular membrane is thought to play a role in high intracellular sodium concentrations.^37^

Children with SAM are more likely to have hyponatremia than are well-nourished children.^8^^,^^38^^,^^39^ Prevalence of hyponatremia upon admission to hospital for children with complicated SAM has been reported to be high, ranging from 21% to 52%.^8^^,^^40–42^ Some studies have reported higher rates of hyponatremia in children with complicated vs uncomplicated SAM, nutritional edema compared with severe wasting, and, in severe malnutrition, with concurrent diarrhea or pneumonia.^40^^,^^43^ Other studies reported similar baseline rates of hyponatremia in complicated and uncomplicated SAM and those with and without diarrhea.^39^^,^^44^

Although hypernatremia has been described in children with SAM, its prevalence is likely lower, ranging from less than 5% to 15% in children with complicated SAM.^8^^,^^40^ The Bandsma et al^8^ study of almost 700 children admitted with SAM in Malawi and Kenya showed that the presence of hypo- or hypernatremia did not change significantly after 3 days of F-75 therapy. To our knowledge, no studies have reported on the continued presence or resolution of hypo- or hypernatremia further into the treatment of SAM.

Previously, hyponatremia was shown to be an independent predictor of death, with studies showing a case fatality rate of 5%–10% in children with diarrhea and hyponatremia.^45^^,^^46^ Another study showed the same mortality rates in malnourished children with or without hyponatremia.^43^ No studies, to our knowledge, have reported on the presence of clinical effects of identified hypo- or hypernatremia specifically in children with SAM. A retrospective study of more than 1200 children under 5 years of age admitted to hospital in Bangladesh showed that children with altered serum sodium levels, including both hypo- and hypernatremia, more often presented with convulsions, altered mental status, dehydration, septic shock, and pneumonia.^46^ In addition, children with hyponatremia were more likely to present at an older age and with SAM.^46^

The major challenge with treating hyponatremia is the widely held belief that treating children with SAM with fluids or foods containing excess sodium may result in increased risk for death, particularly for those with nutritional edema, who are thought to be at risk of cardiac failure and sodium overload.^36^^,^^38^^,^^46^ WHO recommends a solution known as Rehydration Solution for Malnutrition (ReSoMal) or half-strength, standard, low-osmolarity oral rehydration solution for the treatment of dehydration and diarrhea in complicated SAM and avoidance of using intravenous fluids unless in the setting of shock with severe dehydration.^15^ ReSoMal has a sodium content of 45 mmol/L Na, compared with that of the standard WHO low-osmolarity oral rehydration salts, which is 75 mmol/L. The exception to this is in the setting of children with both SAM and cholera or profuse watery diarrhea. These children usually loose high quantities of sodium in their stools and thus should receive the standard WHO low-osmolarity oral rehydration salts.^15^ Recent systematic reviews, though containing small study numbers, considered these universally used oral and intravenous rehydration guidelines as too restrictive, with a lack of evidence to link liberal fluid administration with death or cardiac failure.^47^^,^^48^ Other research comparing different rehydration strategies for severe and moderate dehydration is ongoing.^49^

**Figure 2:**
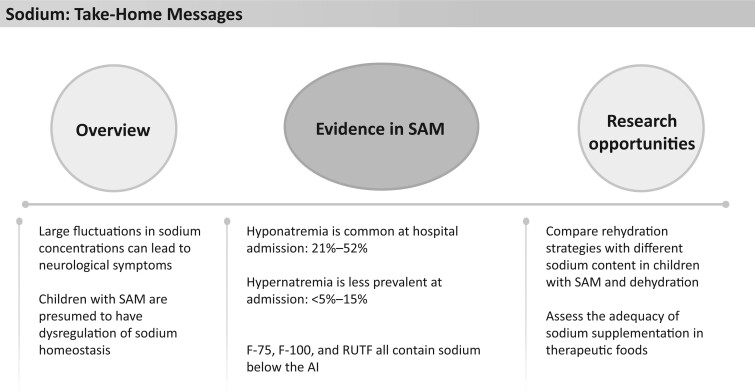
**Sodium take-home messages.** *Abbreviations:* AI, Adequate Intake; RUTF, ready-to-eat food; SAM, severe acute malnutrition.

F-75 also has a low concentration of sodium, containing 7.4 mmol/L (<17 mg/100 mL) and providing just 13%–30% of the AI depending on age. F-100 and RUTF, instead, provide 59%–132% and 35%–78%, respectively, of the AI for sodium (Figure 1, see Tables S4–S12 in the Supporting Information online). Given the high prevalence of hyponatremia and low sodium content of F-75, more targeted studies are needed to decipher whether the current sodium content in therapeutic foods is sufficient to deal with the complex nature of sodium dysregulation in children with SAM. See Figure 2 for sodium take home messages.

### Potassium

**Figure 3:**
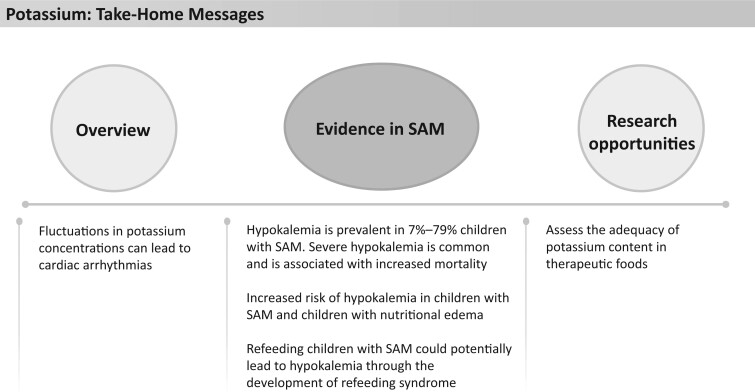
**Potassium take-home messages.** *Abbreviation:* SAM, severe acute malnutrition.

Alteration in serum potassium levels, including both hyper- and hypokalemia, cause hyper- or hypopolarization of the cell, which can result in deadly cardiac arrythmias (Figure 3).^50^ Hypokalemia is defined as a serum potassium level below 3.4 mEq/L in children or below 4.1 mEq/L in infants. Hyperkalemia is defined as a serum potassium level above 4.7 mEq/L in children or above 5.3 mEq/L in infants.^51^

Hypokalemia is a commonly reported electrolyte imbalance in children with SAM, with prevalence rates varying from 7% to 79% in children with complicated SAM.^8^^,^^41^^,^^44^^,^^52^^,^^53^ Only 1 study, to our knowledge, looked at hypokalemia rates between children with complicated (7%; n = 71) and uncomplicated SAM (7%; n = 42) and found no difference.^44^ Though many studies report on levels of hypokalemia, only 1 study could be identified that reported on hyperkalemia. The study authors reported that 30% of children admitted to hospital with complicated SAM had hyperkalemia, and that increased to 61% after 3 days.^8^ It is important to realize that falsely high potassium concentrations are commonly reported in the context of hemolysis during blood sample collection, so the reported prevalence could be an overestimation. Diarrhea in children with SAM likely increases the risk of hypokalemia,^39^^,^^41^^,^^44^^,^^54^ as does the nutritional edematous rather than the wasting phenotype of SAM.^40^^,^^55^ Severe hypokalemia (<2/5 mEq/L) is reported in 22%–30% children with SAM at hospital admission,^52^^,^^56^ with children often having associated electrocardiogram abnormalities, including T-wave inversion.^56^

Hypokalemia is widely accepted as an important cause of death in children with SAM,^35^^,^^52^ with severe hypokalemia reported to have an associated mortality rate of 12%–14%, compared with a mortality rate of 3%–7% in children with normal potassium levels.^52^^,^^53^ Though a drop in phosphate level is the hallmark electrolyte change in refeeding syndrome, hypokalemia is also commonly observed. One Kenyan study of 160 children with SAM^57^ reported 21% of children experienced refeeding syndrome; of these, almost half (n = 16) had hypokalemia on day 2 of feeding. Hypokalemia persisted on day 7 of feeding in 18% (n = 3) of these patients.^57^ A study of 700 children with complicated SAM reported hypokalemia in 23% on admission vs only 4.6% on day 3 of admission.^8^

Current WHO recommendations are that children with SAM and dehydration are treated with low supplementation of sodium and high supplementation of potassium (in the form of ReSoMal), particularly in the setting of diarrhea.^15^ This higher concentration of potassium is thought to promote renal excretion of excess sodium and easing of edema^58^ with increasing serum potassium levels. An older study from Malawi showed that children with nutritional edema who were supplemented with high levels of potassium (8 mmol/kg compared with 4 mmol/kg) had lower mortality rates and improved morbidity.^59^ Therapeutic foods provide potassium at varying concentrations. F-75 contains 142 mg/100 mL potassium, providing 37%–107% of the AI, depending on age. F-100 and RUTF have more potassium: 228 mg/100 mL, providing 80%–232% of the AI or 1171 mg/92 g sachet providing 82%–238% of the AI, respectively, depending on age (Figure 1, see Tables S4–S12 in the Supporting Information online). With hypokalemia being common in children with severe malnutrition, it remains unclear whether the amount provided, especially in F-75, is sufficient to restore and maintain normokalemia. See Figure 3 for potassium take-home messages.

### Calcium

**Figure 4:**
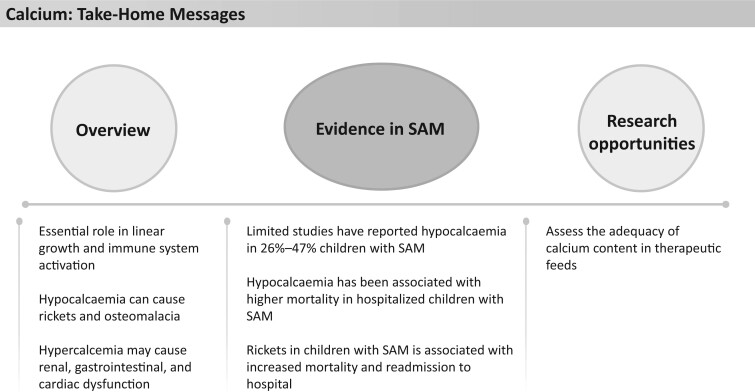
**Calcium take-home messages.** *Abbreviation:* SAM, severe acute malnutrition.

Calcium is essential for linear growth and also plays a central role in the activation of the immune system in response to infection. Hypocalcemia is defined as a serum calcium level less than 8.8 mg/dL (2.2 mmol/L) or an ionized calcium level, its biologically active form, of less than 4.6 mg/dL (1.15 mmol/L). Though clinical characteristics of hypocalcemia are often subtle, including increased neuromuscular irritability, 1 main clinical concern is that it can cause rickets and osteomalacia. Hypercalcemia, which is less common, is defined as a serum calcium level greater than 10.5 mg/dL (2.63 mmol/L) or an ionized calcium level greater than 5.3 mg/dL (1.33 mmol/L).^28^ Hypercalcemia can lead to poor muscle tone, renal insufficiency, hypophosphatemia, constipation, nausea, weight loss, fatigue, polyuria, heart arrhythmias, and a higher risk of cardiovascular disease–related death (Figure 4).^28^

A few cross-sectional studies have reported the prevalence of hypocalcemia in severely malnourished children. Two studies, involving 150 and 333 children, reported hypocalcemia in 26% children admitted with complicated SAM.^60^^,^^61^ A smaller study (n = 120) in Nepal found a higher incidence of hypocalcemia in the malnourished group: 47% compared with 7% in the control group.^62^ No data exist on the prevalence of hypercalcemia in children with severe malnutrition. Hypocalcemia is associated with higher mortality (17%) in hospitalized children with severe malnutrition compared with children without hypocalcemia (5%).^60^ In a study of 150 children with SAM, 42% of children with hypocalcemia had rickets.^61^ In a study of 1778 children with severe malnutrition, rickets was associated with increased mortality, any readmission, and readmission for severe pneumonia.^63^ Although children with SAM have impaired gut function, their calcium absorption efficiency was reported to be comparable to that of healthy children in 1 study.^64^ A high phytate diet and its inhibitory impact on calcium absorption is an important consideration during the recovery from SAM where RUTF may be supplemented with a largely plant-based diet, a diet common in Africa in particular.^65^ On the other hand, the lactose content in F-75 milk and skim-milk–based RUTF could promote calcium absorption.^66^

F-75, F-100, and RUTF provide up to 86%–193%, 114%–256%, and 91%–203%, respectively, of the calcium AI, depending on the age of the child; however, none of these therapeutic foods exceed the UL (Figure 1, see Tables S4–S12 in the Supporting Information online). No data exist, as far as we are aware, on the resolution or persistence of hypocalcemia after nutritional rehabilitation in children with severe malnutrition. The limited evidence on calcium in children with SAM highlights a gap in current knowledge of calcium adequacy in therapeutic foods. See Figure 4 for calcium take-home messages.

### Phosphorus

**Figure 5:**
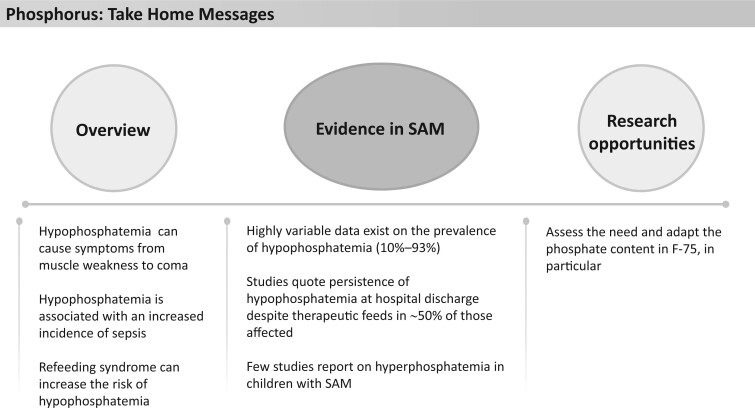
**Phosphorus take-home messages.** *Abbreviation:* SAM, severe acute malnutrition.

Phosphate is important for cellular function and bone mineralization (Figure 5). The reference range for normophosphatemia varies with age, and different laboratories quote different ranges on the basis of the assessment technique. Hypophosphatemia, defined in children as a serum phosphate level less than 4.5 mg/dL (1.45 mmol/L),^51^ can lead to symptoms ranging from muscle weakness to coma and has been associated with an increased incidence of sepsis.^29^ Hyperphosphatemia, defined as a serum phosphate level of greater than 6.5 mg/dL (2.1 mmol/L),^51^ rarely produces adverse effects in healthy people.^29^

The reported prevalence of hypophosphatemia among severely malnourished children admitted to hospital varies widely between studies; different definitions of normal cutoffs likely contribute to this. One large study of almost 700 children in Kenya and Malawi reported hypophosphatemia (phosphate < 0.7 mmol/L) in 10% of children admitted with SAM.^8^ This cutoff level is far lower than that used in 4 smaller studies (phosphate range, <1.1 mmol/L to <1.6 mmol/L) reporting rates of 37%–93% in hospitalized cohorts of 48–120 children with SAM.^67–70^ Two of these smaller studies^67^^,^^70^ divided hypophosphatemia into mild and moderate-severe categories, with prevalence rates of moderate-severe hypophosphatemia of 20%–25%, closer to that reported by Bandsma et al in Kenya and Malawi.^8^

Hypophosphatemia was associated with the presence of hypothermia, skin manifestations, hypoglycemia, hypocalcemia,^67^ hypomagnesemia, and hypoalbuminemia,^68^ and is common in children with sepsis.^70^ The risk of hypophosphatemia can increase upon the initiation of feedings and onset of refeeding syndrome. Studies reported a fall in serum phosphate levels on days 2 and 3 of feedings, particularly if they are not supplemented with phosphate.^42^^,^^67^^,^^71^ In a study predating therapeutic foods, authors quoted 3 weeks to regain a normal phosphate level with nonphosphate-fortified, milk-based diets for malnourished children.^72^ Though levels improve over the course of hospital-based nutritional rehabilitation with F-75 and F-100, hypophosphatemia has been reported to persist in approximately 50% of patients with SAM at discharge from hospital.^68^ However, the mean serum phosphate level at discharge in that study was 1.41,^68^ which, although below the age-specific cutoff for this study, would not be counted as hypophosphatemia in other studies. Bandsma et al^8^ reported the rate of hypophosphatemia in their study of almost 700 severely malnourished children fell from 10% to 5.9% on day 3 of F-75 feeding. Namusoke et al^69^ showed that, with standard nutritional therapy, the mean phosphate level increased from admission through to discharge, with 6% of study participants having persistent hypophosphatemia at discharge and the majority (79%) experiencing their lowest phosphate level at admission. There are far fewer data on hyperphosphatemia in severely malnourished children. One study showed a 30% prevalence of hyperphosphatemia (defined by the authors as >1.5 mmol/L) on admission, which increased to 52% on day 3 of F-75 feeding.^8^

Formulas including F-75, F-100, and RUTF provide approximately 131%–185%, 174%–247%, and 157%–224%, respectively, of the AI for phosphorous, depending on age (Figure 1, see Tables S4–S12 in the Supporting Information online). A summary of the limited data suggests this may be sufficient to prevent clinically relevant hypophosphatemia in most cases. See Figure 5 for phosphorous take-home messages.

### Magnesium

**Figure 6:**
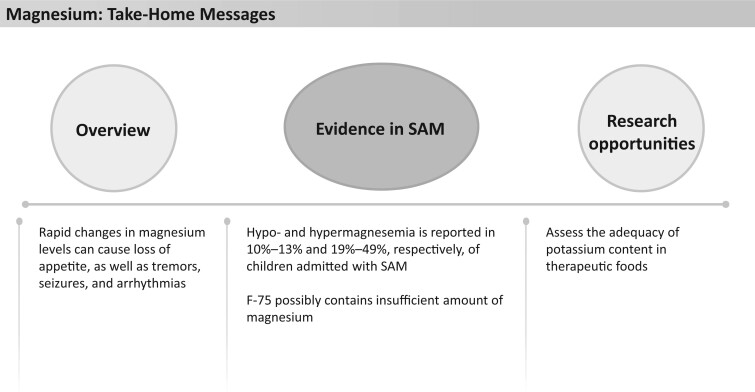
**Magnesium take-home messages.** *Abbreviation:* SAM, severe acute malnutrition.

Magnesium is required for more than 300 enzymatic reactions in the body. Hypo- and hypermagnesemia (serum level <0.7 mmol/L and >0.95 mmol/L, respectively) may be asymptomatic or symptomatic. A sudden change in magnesium levels can result in loss of appetite, tremors, seizures, and arrhythmias (Figure 6).^73^

Varying levels of serum magnesium are found in children with SAM. An Indian study of 43 children reported normal levels upon admission to hospital,^74^ whereas other larger studies, including 72 and 683 severely malnourished children in Africa, reported hypo- and hypermagnesemia in 10%–13% and 19%–49%, respectively, of children admitted with SAM.^8^^,^^68^ An Ethiopian study of 72 children admitted to hospital with SAM showed that admission serum phosphate levels were positively associated with serum magnesium levels, and both were strongly positively correlated with serum albumin levels.^68^ No significant difference was found between children with or without edema.^68^

F-75, F-100, and RUTF are all fortified with magnesium, providing 82%–131%, 245%–393%, and 187%–300%, respectively, of the AI or RDA for magnesium (Figure 1, see Tables S4–S12 in the Supporting Information online). The effect of these therapeutic foods on magnesium levels varies in different studies. A large study of 683 children with SAM reported rates of hypomagnesemia and hypermagnesemia at admission were 10% and 19%, respectively, which changed after 3 days of F-75 therapy to 17% and 9.8%, respectively.^8^ This study would suggest additional magnesium may be required in F-75 to maintain a normal magnesium status. However, a smaller (n = 72), though longer, study showed that when F-75 or diluted F-100 (used due to inadequate F-75 stock) was administered to patients with SAM, a subsequent increase in magnesium occurred, with all patients having normal or high (83%) concentrations of magnesium at discharge from hospital, indicating sufficient supplementation.^68^ An additional study investigating the effect of combined intramuscular (day 1) and oral magnesium (days 2–14) supplementation on top of therapeutic feedings in 50 children with SAM in India found a reduction in magnesium level from admission to discharge, though all levels were within the normal range.^74^ More studies are required to determine the adequate magnesium dose in therapeutic foods, in particular, F-75. See Figure 6 for magnesium take-home messages.

### Iron

**Figure 7:**
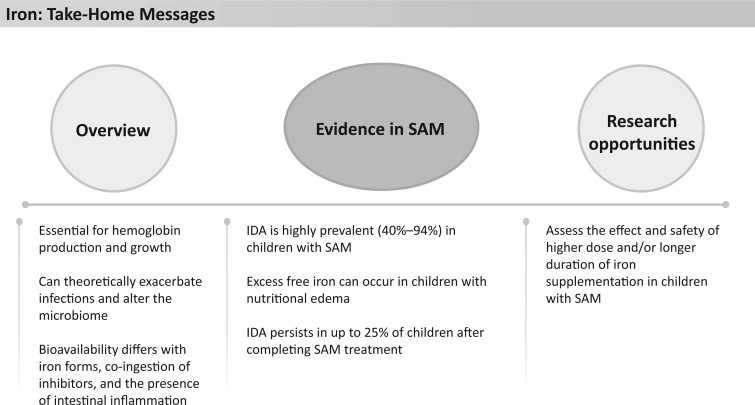
**Iron take-home messages.** *Abbreviations:* IDA, iron deficiency anemia; SAM, severe acute malnutrition.

Iron is a central component of hemoglobin and red blood cell production, and is involved in many enzymatic reactions. The main concern with iron deficiency is the development of iron deficiency anemia (IDA). Anemia, defined as a hemoglobin concentration of less than 110 g/L (severe anemia, <70 g/L), can lead to fatigue, weakness, difficulty concentrating, and impaired immune function and may cause irreversible growth and cognitive delays if left untreated.^75^ Though the prevalence of IDA has declined slightly worldwide over the past 25 years, it continues to affect close to 300 million children, mostly in LMICs where SAM is also highly prevalent (Figure 7).^75^^,^^76^

Several studies have reported that children with SAM present with high rates of IDA. In a cohort of 801 children in Burkina Faso, IDA rates on admission were 40%,^77^ and higher rates were observed in multiple smaller trials conducted in Malawi and India, with sample sizes of 389 (48% IDA), 131 (67% IDA), and 50 (94% IDA) children with SAM.^78–80^ Additionally, 2 of these studies reported that 25%–46% of patients had anemia categorized as severe^79^^,^^80^ and required transfusion.^79^ Conversely, some studies found that children with nutritional edema present with increased serum free iron, compared with children with wasting or not, and researchers have suggested this represents iron overload.^81–85^

Currently, F-75 contains very low levels of iron, providing between 4% and 9% of the RDA (Figure 1, See Tables S4–S6 in the Supporting Information online). Given that F-75 is provided for stabilization in the early stages of treatment, this is likely appropriate because iron supplementation, theoretically, may exacerbate infections^86^; thus, low iron doses may mitigate this risk. However, F-100 also provides considerably low amounts of iron, meeting only 6%–15% of the RDA, despite its role in rehabilitation (Figure 1, See Tables S7–S9 in the Supporting Information online). Although most patients will also only use F-100 for a short time, those with concurrent SAM and swallowing dysfunction, such as patients with cerebral palsy, may require F-100 for the duration of their rehabilitation. On the other hand, RUTF contains much higher amounts of iron and provides between 163% and 441% of the RDA (Figure 1, See Tables S10–S12 in the Supporting Information online). Although this may seem excessive, iron needs are likely increased due to increased growth velocity.^77^ Additionally, although most children with SAM complete treatment within 6–8 weeks, IDA likely remains prevalent, with rates reported to be 21%–25% among children discharged from treatment despite meeting growth requirements for discharge.^77^^,^^78^ One study compared 2 alternative, plant-based RUTF products with lower concentrations of cow’s milk (0% and 9% milk) than standard RUTF (28% milk) and showed considerably lower rates of IDA upon completion of treatment of the plant-based RUTF (12%–18% vs 25% in those receiving standard RUTF).^78^ The authors attributed the improvement in IDA rates to the lower amount or absence of cow’s milk in the recipes and subsequent improved bioavailability of iron and reduction of potential iron inhibitors, namely, calcium, whey, and casein.^78^ However, other experts have also noted the considerably higher amounts of iron (non-heme compared with heme iron in standard RUTF) in the alternative RUTF products (35.1 mg/100 g in 0% milk and 31.6 mg/100g in 9% milk)) compared with standard RUTF (10.5 mg/100g).^77^^,^^82^

It is possible that malnutrition-associated enteropathy and inflammation may upregulate the regulatory protein hepcidin, thereby decreasing iron absorption from the enterocyte by trapping the iron in the enterocyte, as has been shown in inflammatory bowel disease and other conditions.^87^^,^^88^ For this reason, higher-dose enteral iron supplementation may not result in increased iron levels until inflammation is controlled; instead, intravenous iron supplementation or longer durations rather than higher doses of enteral iron supplementation may be physiologically better therapeutic options.^89^

Interestingly, novel nano-iron supplements have recently been shown to improve iron stores while reducing moderate-severe diarrhea compared with standard ferrous sulphate iron supplements.^90^ These supplements may be a potential treatment consideration in the future. It may be valuable to explore whether increased amounts of iron in F-100, and possibly RUTF; improvement in bioavailability of iron; longer duration of iron supplementation beyond standard nutritional therapy; or intravenous iron supplementation could lead to improvement in iron status in children recovering from SAM. However, these efforts must be balanced with the potential risks of additional iron, including possible negative effects on the microbiota, impacts on growth (particularly in iron-replete children), impairment of cognitive development, and increased gastrointestinal side effects resulting in nonadherence and interference with absorption of other trace elements.^77^^,^^86^ See Figure 7 for iron take-home messages.

### Zinc

**Figure 8:**
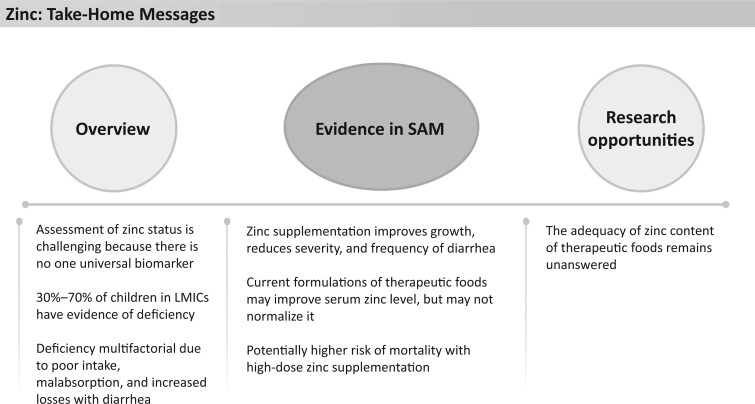
**Zinc take-home messages.** *Abbreviations:* LMIC, lower- and middle-income country; SAM, severe acute malnutrition.

Clinical features of zinc deficiency include poor appetite, growth failures, skin lesions, diarrhea, poor wound healing, and impaired immune response. Assessment of zinc status is challenging given that there is not 1 widely accepted sensitive and specific biomarker of zinc status, and zinc status is affected by multiple factors, including the acute-phase response in infection.^91^ The prevalence of zinc deficiency is estimated to include 30%–70% of children in LMICs and is likely related to a combination of factors, including poor intake, malabsorption, or increased losses due to diarrhea (Figure 8).^92–95^ Multiple studies have shown that children with malnutrition had significantly lower levels of serum zinc than did healthy children.^95–97^ Studies have indicated clinical benefits of zinc supplementation, such as improved growth and reduction of both acute and chronic diarrhea.^98–101^ WHO advises 14 days of supplemental zinc at 20 mg/d for children with acute diarrhea to reduce diarrhea frequency and severity.^102^

Current SAM treatment protocols provide between 16 and 36 mg/d zinc depending on the phase of treatment and age (Figure 1, see Tables S4–S12 in the Supporting Information online). This exceeds the UL for zinc in all cases (5–7 mg/d depending on age). However, it should be noted that the UL for zinc has also been criticized for being too low,^103^ and it has been suggested that zinc requirements for children in LMICs may be increased because of the high levels of phytate in cereal-based diets and the poor access to meats and shellfish.^94^ A study in India reported that a combined supplemental antioxidant (zinc dose not reported) for 30 days significantly increased and normalized serum zinc levels in malnourished children, though it must be noted their nutritional intake was otherwise not commented upon.^97^ One study involving 12 patients with SAM in Bolivia^104^ found that serum zinc status improved on the WHO SAM protocol, but that it may take up to 30 days of treatment before normalization of serum zinc levels. It is notable that up to 25% of the patients in that study did not have improvements in serum zinc status,^104^ suggesting insufficient supplementation in therapeutic food products. However, higher doses of zinc supplementation (6 mg/kg/d) started early in treatment (day 1 of admission) and lasting for 15–30 days have been linked to higher rates of mortality than in children receiving lower doses of supplementation (1.5 mg/kg/d = 7.5–12.75 mg/d).^105^ It is thought that the initiation of high doses of zinc during stabilization may have harmful effects on the immune response during sepsis and affect absorption of other micronutrients.^105^^,^^106^ More investigation into the optimal intake of zinc in children with SAM is warranted. See Figure 8 for zinc take-home messages.

### Copper

**Figure 9:**
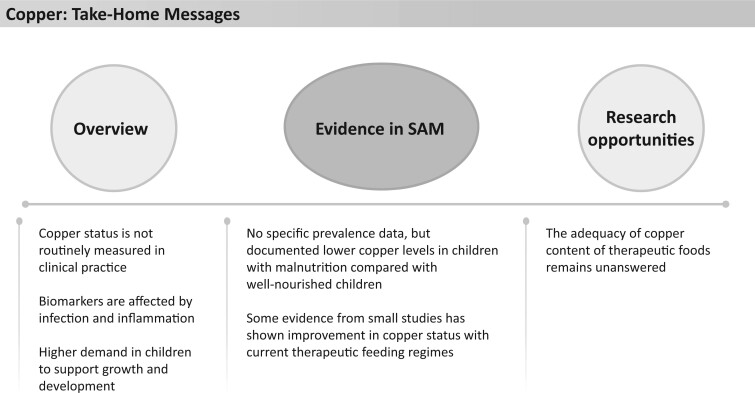
**Copper take-home messages.** *Abbreviation:* SAM, severe acute malnutrition.

Copper status is not routinely assessed in clinical practice, and the available biomarkers (serum copper and ceruloplasmin) to assess status can be influenced by other factors, such as infection and inflammation (Figure 9).^107^ In children and infants, the demand for dietary copper is higher than in adults for their development and growth.^108^

Copper deficiency may lead to the development of muscle numbness, burning, optic neuropathy, fatigue, weakness, IDA, metabolic syndrome, and increased risk of infection. Copper toxicity is rare in individuals who do not have a hereditary copper homeostasis defect but can lead to liver disease, gastrointestinal symptoms, hemolytic anemia, anuria, and central nervous system symptoms, such as motor and cognitive abnormalities.^32^

As with other trace elements, children with malnutrition are at risk of acquired copper deficiency. Several studies based on cohorts from India (n = 117),^95^ Bangladesh (n = 120 and n = 68),^96^^,^^109^ Turkey (n = 32),^110^ Egypt (n = 68),^81^ Morocco (n = 36),^111^ and Tanzania (n = 50)^112^ documented lower copper levels in severely malnourished children compared with healthy, well-nourished children. One study in India reported copper deficiency only in malnourished children with marked linear growth retardation.^113^ Therapeutic foods contain high amounts of copper and exceed the UL in all products for children older than 1 year (Figure 1, see Tables S4–S12 in the Supporting Information online). There is no UL available for younger children; however, all products exceed the AI for this age group. Small studies in South Africa (n = 22) and Bolivia (n = 12) showed that WHO nutritional therapy could return serum copper and ceruloplasmin levels to normal within 15 days^104^ to 30 days,^114^ together with significant weight gain.^114^ Neither toxic levels nor adverse events were reported in these studies.^104^^,^^114^ In summary, there are some small, underpowered studies that suggest that current amounts of copper provided in therapeutic foods are sufficient to address copper deficiency in severely malnourished children. However, larger scale studies would be required to verify this. See Figure 9 for copper take-home messages.

### Selenium

**Figure 10:**
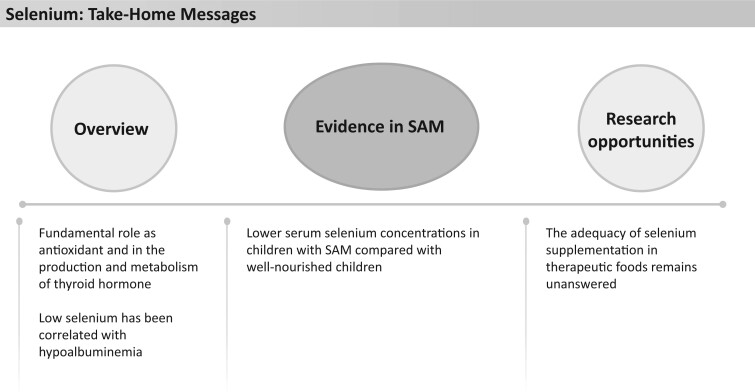
**Selenium take-home messages.** *Abbreviation:* SAM, severe acute malnutrition.

Selenium is fundamental for human health through its antioxidant properties to prevent oxidative damage by free radicals and its role in the production and metabolism of thyroid hormone (Figure 10). Selenium status is generally reported as concentration is serum, which is indicative of recent selenium intake, whereas hair or nail selenium levels are better indicators of longer-term intake over months to years.^30^ It is worth noting that a study in infants showed a correlation between low selenium levels and hypoalbuminemia, recommending both levels be assessed when interpreting possible deficiency.^115^ Selenium deficiency presents clinically in the form of cardiomyopathies (Keshan disease), skeletal muscle myopathies (Kashin–Beck disease), and microcytic anemia. Growth retardation and alopecia with pseudoalbinism are early clinical symptoms of deficiency in infants, which are reversible with adequate amounts of selenium intake.^116^

Previous theories of a causative association between oxidative stress and the edematous phenotype of SAM^117^ were refuted in a double-blind, randomized controlled trial (RCT; n = 2372) in Malawi in which daily high-dose antioxidant supplementation including selenium for 5 months did not prevent the onset of nutritional edema in children.^118^ Multiple studies in Egypt (n = 68), Morocco (n = 160), South America (n = 43), Sudan (n = 53), and Turkey (n = 32) found that lower serum selenium concentrations were present in children with nutritional edema compared with children who had severe wasting and in children with severe malnutrition compared with healthy, well-nourished children from the same communities.^81^^,^^110^^,^^111^^,^^114^^,^^119–121^

Studies looking at the outcomes of selenium deficiency in children with SAM are sparse. One study of more than 500 children in Ethiopia found that selenium deficiency (measured by serum levels of selenium) was associated with lower scores for all cognitive outcomes on the Wechsler Preschool and Primary Scale of Intelligence and a 25-item school-readiness test, compared normal serum selenium levels in children.^122^ There was no significant difference in selenium level between children with stunting and those without stunting. However, it was reported that children with coexisting stunting, anemia, iron deficiency, and selenium deficiency scored significantly lower on these cognitive tests than those without these burdens.^122^ Selenium content in F-75, F-100, and RUTF is approximately 1.6–4.3 times greater than the AI or RDA for children aged 6–59 months; however, it is below the UL for each age group (Figure 1, see Tables S4–S12 in the Supporting Information online). A small study in 1992 showed that milk intervention in children aged 5–48 months (n = 22) with nutritional edema improved plasma selenium concentration from baseline but not to levels of well-nourished healthy community control participants (n = 22) at 30 days of refeeding.^114^ Interestingly, a study of 160 children with SAM in Egypt showed resolution of selenium deficiency at the end of WHO-recommended nutritional therapy for all neurotypical children, though deficiency persisted for a large proportion of children with both SAM and cerebral palsy.^119^ The result of selenium supplementation on developmental outcomes in children with SAM has not been assessed in controlled trials, to our knowledge. Although no direct evidence exists, it is possible that selenium deficiency is common in children with severe malnutrition and may be improved during nutritional rehabilitation with WHO-recommended therapies; however, it remains unclear if the deficiency is fully corrected with these approaches. See Figure 10 for selenium take-home messages.

### Iodine

**Figure 11:**
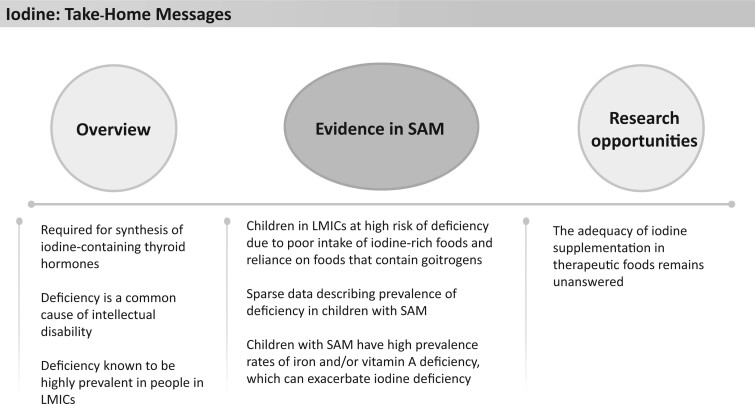
**Iodine take-home messages.** *Abbreviations:* LMIC, lower- and middle-income country; SAM, severe acute malnutrition.

Iodine is required for the synthesis of iodine-containing thyroid hormones, thereby helping in the regulation of the basal metabolic rate. Iodine deficiency is considered a common cause of intellectual disability and can lead to cognitive and motor impairments (Figure 11). A clinical sign of iodine deficiency is the development of a goiter.^30^^,^^123^ Iodine status is biochemically measured using urinary concentrations of iodine, with below 100 µg/L considered to be inadequate and below 20 µg/L considered severely deficient.^30^^,^^123^^,^^124^

Iodine deficiency is highly prevalent among people in LMICs, affecting 1.88 billion people worldwide, including 241 million school-aged children, with many regions considered to have endemic iodine deficiency.^125^ Many children in LMICs are at increased risk of developing iodine deficiency due to poor intake of iodine-rich foods and the reliance on food that contain goitrogens, such as soy, cassava, and cruciferous vegetables, which may exacerbate iodine deficiency.^30^^,^^124^^,^^126^ For these reasons, the risk of iodine deficiency for children with SAM is likely high; however, there are little to no data describing prevalence of iodine status in this population. Some small studies (n = 12–68) suggest that intestinal dysfunction in children with SAM may contribute to malabsorption of iodine.^127–130^ To our knowledge, however, there are no reports on urinary iodine concentrations in these children. Additionally, children with SAM high prevalence rates of iron and/or vitamin A deficiency (VAD), which are also known to exacerbate iodine deficiency.^30^^,^^124^^,^^126^

The current F-75 content of iodine is 90% and 220% of the AI and RDA for children aged 6–12 months and12–23 months, respectively; whereas F-100 and RUTF both provide higher amounts: 135% and 333% (F-100) and 132% and 327% (RUTF). It must be noted that the iodine content of both F-100 and RUTF exceed the UL recommended for children older than 1 year (the UL is not established for children 6–12 months old) (Figure 1, see Tables S4–S12 in the Supporting Information online). In other populations, it has been shown that providing supplemental iodine can be associated with transient hyperthyroidism^124^; however this has not been explored in children with SAM, despite intakes exceeding the UL with standard nutritional therapy. Given the importance of iodine on cognitive development, it may be valuable to assess prevalence of iodine deficiency in children with SAM and subsequent response to therapeutic feedings. See Figure 11 for iodine take-home messages.

### Vitamin A

**Figure 12:**
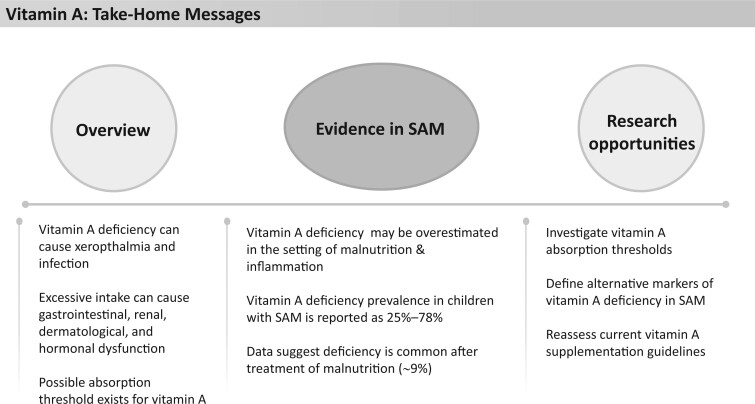
**Vitamin A take-home messages.** *Abbreviation:* SAM, severe acute malnutrition.

Vitamin A is essential in growth, reproduction, immunity, vision, and, more broadly, cellular differentiation and proliferation (Figure 12).^131^ The main clinical manifestations of VAD are xeropthalmia and increased susceptibility to severe infection. Systemic vitamin A toxicity can be due to acute or chronic excessive ingestion, and its effects include altered mucocutaneous abnormalities, headaches, nausea, vomiting, skin irritation, and reversible hypothyroidism and renal dysfunction. The adverse effects seen with single high-dose vitamin A supplementation are usually mild and transient, and include nausea, vomiting, headaches (in older children), and bulging fontanelles (in younger children).^132^

VAD is commonly defined as plasma or serum retinol concentration or retinol binding protein (RBP) level less than 0.70 μmol/L.^133^ However, serum retinol levels only reflect liver vitamin A stores when they are extremely depleted or high. Additionally, RBP, which is produced by the liver, can be reduced in malnutrition and inflammation, thereby overestimating VAD.^134^ Liver vitamin A reserves, a better reflection of subclinical VAD, can be measured indirectly with a relative dose–response test, but this is more time consuming and costly.^135^ The prevalence of VAD globally has reduced from 39% to 30% over the past 2 decades (1991–2013) with little to no improvement in the prevalence levels seen in sub-Saharan Africa and South Asia. A pooled analysis of population-based studies estimated approximately 94 500 deaths due to diarrhea and 11 200 deaths due to pneumonia were attributed to VAD globally in 2013, accounting for 1.7% of all deaths in children younger than 5 years in LMICs.^136^ WHO recommends periodic supplementation in areas with high prevalence of night blindness or VAD and acutely in the setting of measles.^132^

Current WHO recommendations for vitamin A supplementation in children with SAM are ∼5000 IU/1500 µg retinol activity equivalent vitamin A daily (1 IU retinol = 0.3 μg retinol activity equivalent), either as an integral part of therapeutic foods or as part of a multi-micronutrient formulation.^15^ F-75, F-100, and RUTF provide 286%–810%, 394%–1125%, and 277%–790% of the AI and RDA requirements for vitamin A, respectively, depending on age, and contain 2.3–5.6 times the reported daily UL, depending on the age group (Figure 1, see Tables S4–S12 in the Supporting Information online).

VAD in children with SAM has been reported to range from 25% to 78%,^77^^,^^137^^,^^138^ though many of these studies did not correct the retinol or RBP level for inflammation. A recent study in Burkina Faso noted 25% of children with uncomplicated SAM had VAD, with levels corrected for inflammation, when admitted to their nutrition program. This percentage decreased to 9% after an average of 2 months of nutritional treatment.^77^ Interestingly, in this study,^77^ researchers compared the effects of reduced-dose RUTF with standard dosing (anything from a 10% to ∼50% reduction in RUTF dose with an equivalent reduction in vitamin A supplementation) and found no difference in VAD between the reduced and standard RUTF dosing groups at the end of the treatment period.^77^ A study in Bangladesh, looking at the safety of a single high dose (200 000 IU) of vitamin A in conjunction with daily low dose (5000 IU) vitamin A in comparison with standard low-dose vitamin A (5000 IU) in children with SAM and diarrhea or lower respiratory tract infection found no difference in efficacy or safety over the 15-day study period.^138^ Though the RBP levels may have been confounded by a concordant country-wide biannual vitamin A supplementation in the Burkina Faso study,^77^ both studies raise questions about possible vitamin A absorption thresholds, vitamin A liver reserves, the appropriateness of using retinol or RBP as a marker of VAD in children with SAM and the possible need for continued micronutrient supplementation after nutritional therapy is completed. More research into these questions is needed before changes to dosing guidelines can be safely made. See Figure 12 for vitamin A take-home messages.

### Vitamin D

**Figure 13:**
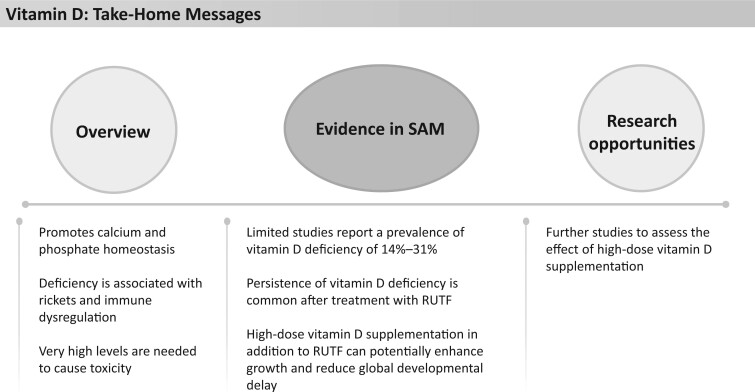
**Vitamin D take-home messages.** *Abbreviation:* SAM, severe acute malnutrition.

The main function of vitamin D in the body is promoting calcium and phosphate homeostasis, thereby helping to maintain skeletal health and prevent rickets in children (Figure 13). Vitamin D deficiency is also associated with adverse respiratory outcomes in asthma exacerbations and tuberculosis reactivation, likely due to an effect on the immune system.^139^ Vitamin D status is indicated by serum levels of 25-(OH)D, though variation in levels exist depending on the assay used.^140^ Though there is no consensus on the optimal level of vitamin D to maintain good bone health, the most commonly used definitions consider concentrations greater than 50 nmol/L (>20 ng/mL) to be normal, 30–50 nmol/L (12–20 ng/mL) to be insufficient, and less than 30nmol/L (<12ng/mL) to be deficient.^141^ The level for vitamin D toxicity is considered to be greater than 375 nmol/L (>150 ng/mL).^142^ Vitamin D toxicity in children is rare but reported among patients taking very high doses of vitamin D. Its adverse effects are attributed to hypercalcemia and include nausea, vomiting, muscle weakness, neuropsychiatric disturbances, polyuria, excessive thirst, and kidney stones.^143^

Though data are scant, a recent review of worldwide vitamin D status suggests widespread vitamin D deficiency, with the highest prevalence in Asia, the Middle East, and Africa. The prevalence of vitamin D deficiency in malnourished children reported in the literature varies widely, from 14% to 31% in children with SAM or moderate acute malnutrition in 2 studies in Africa^144^^,^^145^ to 65% in children with SAM reported in a study in India.^61^ Though immunoassay methods were used to determine 25-(OH)D level in each of these studies, they differed in their definition of deficiency. The reported prevalence of rickets in children with SAM also differs in studies, ranging from 13% in a Kenyan study^63^ to 42% in an Indian study.^61^ Importantly, 1 group in Kenya showed an association between rickets in this population cohort, with an increased risk of mortality, hospital readmission, and readmission with pneumonia.^63^

F-75, F-100, and RUTF therapeutic feedings provide 195%–276%, 301%–430%, and 196%–280%, respectively, of the RDA for vitamin D (Figure 1, see Tables S4–S12 in the Supporting Information online). There is a lack of studies evaluating the exact effect of nutritional treatment of SAM on vitamin D levels. One RCT in Pakistan^146^ of high-dose vitamin D_3_ (2 oral doses of vitamin D_3_ 200 000 IU at weeks 2 and 4) in addition to RUTF (600 IU/sachet) in children with SAM found increased weight-for-height or for length *z* score, increased weight, and reduced rate of global development delay vs RUTF alone. All patients who received additional high-dose vitamin D had normal (>50 nmol/L) 25-(OH)D levels at week 8 (study end) in comparison with only 42% of those treated with RUTF alone. Of note, 27% of patients who received additional high-dose vitamin D had levels exceeding 125 nmol/L but less than 250 nmol/L, and no adverse effects or hypercalcemia were detected.^146^ A larger phase 2 RCT in Pakistan is currently underway aiming to show weight gain (primary outcome) and enhanced neurodevelopment, muscle mass accumulation, resolution of systemic inflammation, and antimicrobial immune function (secondary outcomes) in children with SAM supplemented with high-dose vitamin D.^147^ The results of this study could help guide whether an increased dose of vitamin D supplementation for children diagnosed with SAM is warranted, as expected. See Figure 13 for vitamin D take-home messages.

### Vitamin E

**Figure 14:**
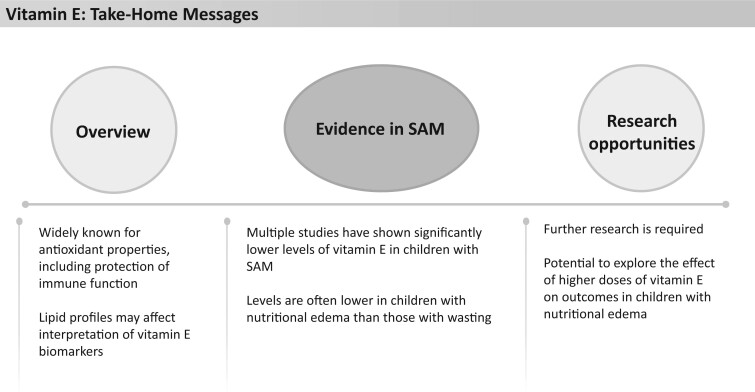
**Vitamin E take-home messages.** *Abbreviation:* SAM, severe acute malnutrition.

Vitamin E is widely known for its antioxidant properties (Figure 14). It protects immune function by serving as an oxidant scavenger and protecting cells from free-radical damage.^148^^,^^149^ Deficiency has been linked to spinocerebellar ataxia, skeletal myopathy, retinopathy, and anemia.^30^^,^^148^ Determination of deficiency is challenging given that plasma levels are affected by confounding factors such as age, sex, and lipid profiles, which may be altered in the context of SAM.^148^ Because α-tocopherol levels are highly correlated with blood lipid levels, ratios of α-tocopherol to plasma lipids are often considered more accurate measures of vitamin E status. However, both of these measures may produce unreliable results.^148^

Multiple studies have shown that children with SAM have both significantly lower serum vitamin E levels and lower α-tocopherol to lipid ratios.^81^^,^^97^^,^^150–153^ Importantly, these levels are often lower in children with nutritional edema than in those with wasting.^81^^,^^151^ Vitamin E deficiency has been proposed as a potential factor in the onset of nutritional edema.^117^ Golden and Ramdath^117^ proposed that nutritional edema results from a leakage in the vascular system when antioxidant activity is not sufficient to neutralize free-radical damage produced by the inflammatory response. However, in a 2005 study, high-dose vitamin E supplementation in 2372 children (given in an antioxidant complex also containing riboflavin, selenium, and *N*-acetylcysteine) in rural Malawi did not improve the likelihood of developing nutritional edema, compared with placebo.^118^

Specifically, F-75, F-100, and RUTF provide 540%–764%, 831%–1187%, and 644%–920%, respectively, of the AI or RDA for vitamin E (Figure 1, see Tables S4–S12 in the Supporting Information online). These supplementation doses clearly exceed the AI or RDA for all age groups but remain far below the UL. However, given the findings that children with nutritional edema typically have lower levels of biomarkers of vitamin E circulating and that these lower levels have the potential to be related to poor immune function, it is worth further investigation to determine if higher doses of supplemental vitamin E in therapeutic feeding regimes would improve the outcomes in children with SAM, particularly nutritional edema. See Figure 14 for vitamin E take-home messages.

### Vitamin K

**Figure 15:**
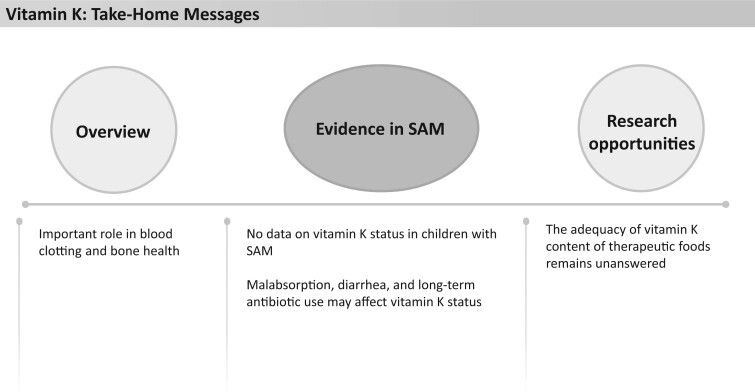
**Vitamin K take-home messages.** *Abbreviation:* SAM, severe acute malnutrition.

Vitamin K status has an important role in blood clotting, and deficiency typically presents with a vitamin K–responsive increase in prothrombin time and, in severe cases, a hemorrhagic event. Vitamin K also plays a role in bone health, including bone mineralization and turnover (Figure 15).

To our knowledge, there are no dedicated data on vitamin K status (namely, intake, requirements, and rates of deficiency) in children with SAM. However, it is well known that malabsorption, diarrhea, and long-term antibiotic use (>10 days) may affect vitamin K status.^32^^,^^154^ One study in India assessed vitamin K status of sick children (n = 120) receiving long-term antibiotic therapy and found a trend in children with severe malnutrition toward increased prothrombin time compared with children with no or moderate malnutrition.^154^ The same study showed that providing a prophylactic dose of vitamin K (0.5 mg/kg to a maximum of 10 mg) on day 1 of antibiotic therapy did not mitigate this effect, indicating factors other than vitamin K deficiency are important.^154^ It is standard practice to initiate antibiotics in children with SAM, which may affect vitamin K status and prothrombin time, but there is limited evidence on this relationship in this population.

F-75, F-100, and RUTF provide 140%–1404%, 215%–2153%, and 101%–1008%, respectively, of the AI or RDA for vitamin K depending on the age of the child (Figure 1, see Tables S4–S12 in the Supporting Information online). No UL is reported for infants and children aged less than 3 years. These high levels may help prevent the development or exacerbation of deficiency during SAM treatment. More studies are required to assess the status of vitamin K in children with SAM and rates of vitamin K deficiency with antibiotic therapy in this population. See Figure 15 for vitamin K take-home messages.

### Ascorbic acid

**Figure 16:**
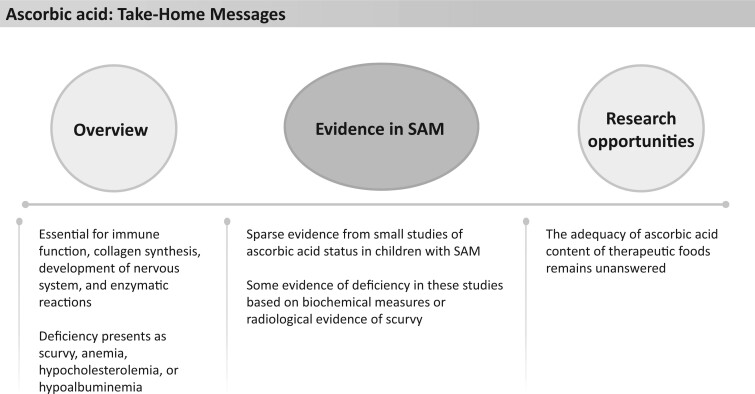
**Ascorbic acid take-home messages.** *Abbreviation:* SAM, severe acute malnutrition.

Ascorbic acid, more commonly known as vitamin C, is essential for immune function, collagen synthesis, and development of the nervous system, and is also a cofactor for enzymatic reactions (Figure 16).^155^^,^^156^ Deficiency can present as scurvy, which is characterized by gingival swelling, corkscrew and swan-neck hair formation, fatigue, muscle pain, joint pain, and bleeding.^30^ It can also present with anemia, hypocholesterolemia, and hypoalbuminemia.^30^ Vitamin C deficiency is diagnosed by serum or plasma levels below 0.2 mg/dL ascorbic acid (the reduced form of vitamin C), though these levels can be affected by recent dietary intake.^157^ A more accurate measure of deficiency is leukocyte ascorbic acid (<8 mg/dL is considered deficient), which is reflective of tissue stores.^158^ However, leukocyte levels can be reduced in certain disease states, including asthma and diabetes, and both serum and leukocyte ascorbic acid levels can be reduced in infection and stress. It is recommended that both levels be measured when assessing vitamin C status.^159^

Both intestinal barrier dysfunction and oxidative stress seen in children with SAM have been postulated to contribute to vitamin C deficiency.^81^^,^^160^ Little is known about vitamin C status in children with SAM. One small study (n = 26) in Nigeria reported similar but low leukocyte ascorbic acid levels (8–15 mg/dL) in children with SAM and healthy children.^161^ A study of 50 children with SAM in India found that 96% of the children had radiological evidence of vitamin C deficiency,^162^ though another Indian study reported that only 5% of children with SAM had cutaneous symptoms of vitamin C deficiency.^163^ F-75, F-100, and RUTF provide 111%–626%, 166%–950%, and 161%–920%, respectively, of the vitamin C AI or RDA; however, none of these therapeutic foods exceeds the UL (Figure 1, see Tables S4–S12 in the Supporting Information online). More studies are needed to ascertain the vitamin C status in severely malnourished children and decipher if the current supplementation in therapeutic foods is safe and adequate. See Figure 16 for ascorbic acid take-home messages.

### Thiamine

**Figure 17:**
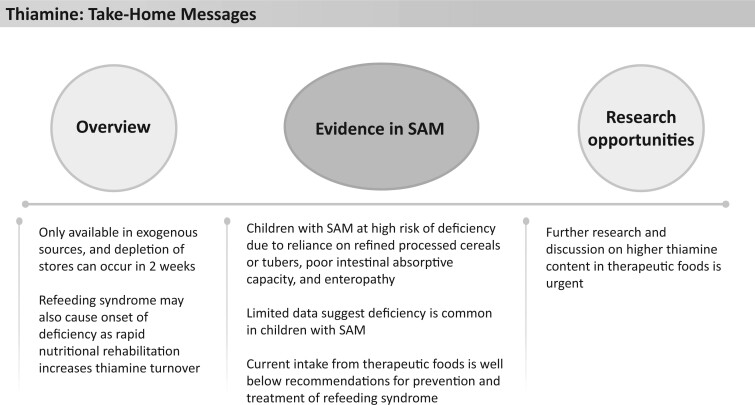
**Thiamine take-home messages.** *Abbreviation:* SAM, severe acute malnutrition.

Thiamine (vitamin B_1_) is an essential nutrient that plays a role in many cellular processes, including carbohydrate and amino acid catabolism (Figure 17). Signs of deficiency include anorexia, weight loss, cognitive changes, muscle weakness, and cardiovascular effects.^31^ Thiamine is only available through exogenous sources, and depletion of stores can occur in 2 weeks.^164^ Refeeding syndrome may also cause the onset of thiamine deficiency because rapid nutritional rehabilitation increases thiamine turnover.^164^ Children in LMICs, particularly children with SAM, are at high risk of deficiency for several reasons, including their reliance on refined processed cereals or tubers (eg, rice, wheat, cassava), poor intestinal absorptive capacity, and enteropathy.^31^^,^^164^^,^^165^ Toxic effects have not been demonstrated with higher doses of thiamine.^31^^,^^165^ For this reason, it has been recommended to treat patients at risk of refeeding syndrome with high doses of thiamine (recommended range: 1–2mg/kg/d to 100–300 mg/d) to prevent the onset of thiamine deficiency with the provision of nutrition.^166–168^

Studies in Ghana (n = 146) and Jamaica (n = 25) have indicated a prevalence of 40% of children with SAM having moderate to severe thiamine deficiency upon hospital admission.^169^^,^^170^ Furthermore, the Ghanaian study indicated that deficiency resolved in children who were treated with 2.5 mg/d thiamine.^170^ Currently, F-75 provides between 0.55 and 0.94 mg/d, which is significantly lower than the recommendations cited above for refeeding risk (Figure 1, see Tables S4–S6 in the Supporting Information online). Hiffler et al^164^^,^^165^ cited the low risk of side effects with high doses of thiamine, the high prevalence of thiamine deficiency in children with SAM, and the potential to improve outcomes with higher doses of thiamine during stabilization as rationales for increasing the amount of thiamine in F-75 from 0.085 mg/100 mL to 7.5 mg/100 mL, which would provide patients between 50 and 80 mg/d thiamine. Furthermore, given that thiamine stores are directly related to recent intake, children with SAM may benefit from higher doses of thiamine in both F-100 and RUTF than what is currently provided. F-100 and RUTF provide between 0.1 and 1.4 mg/d, depending on age (Figure 1, see Tables S7–S12 in the Supporting Information online). It has been suggested that 5–10 mg/d during nutritional rehabilitation would be appropriate.^164^ Additionally, F-100 and RUTF could be increased to 1.0 mg/100 mL and 4.5 mg/92 g, respectively, to provide the recommended 1–2 mg/kg/d for children.^164^^,^^166^ The current evidence strongly supports that research and discussion on providing a higher content of thiamine in therapeutic foods are urgent. See Figure 17 for thiamine take-home messages.

### Riboflavin

**Figure 18:**
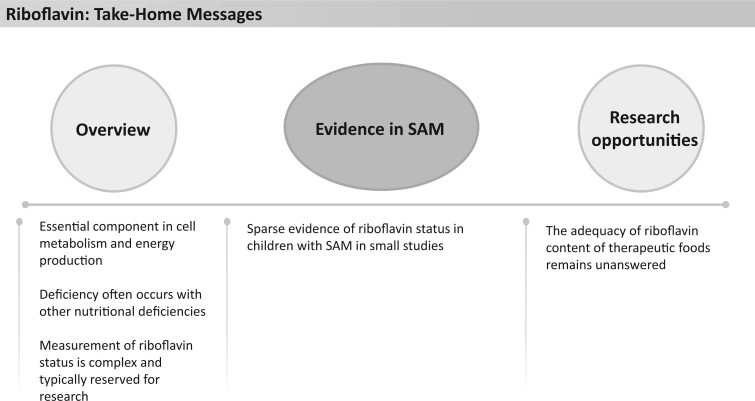
**Riboflavin take-home messages.** *Abbreviation:* SAM, severe acute malnutrition.

Riboflavin (vitamin B_2_) is an essential component of 2 coenzymes, flavin mononucleotide (FMN) and flavin adenine (FAD) that are involved in cell metabolism and energy production (Figure 18). Deficiency of riboflavin typically occurs in conjunction with other nutritional deficiencies, and symptoms of deficiency may include skin disorders, sore throat, increased blood volume, glossitis, angular stomatitis, and anemia.^31^^,^^123^ Riboflavin deficiency is determined by measuring concentrations of coenzymes FMN and FAD or by measuring erythrocyte glutathione reductase activity coefficient (EGRAC), which is a ratio of erythrocyte glutathione reductase activity in the presence of FAD to that without added FAD. A ratio greater than 1.4 indicates riboflavin deficiency.^31^

Evidence of riboflavin status in children with SAM is sparse. Three small studies reported no evidence of riboflavin deficiency at presentation in children with SAM. However, 1 study in Nigeria noted that although children with SAM (n = 44) did not have evidence of riboflavin deficiency based on EGRAC, control study participants (n = 23) did.^171^ This finding could be related to muscle breakdown releasing FAD, which can normalize intracellular FAD levels, including in erythrocytes.^171^^,^^172^ Additionally, a study from western Africa^173^ examined riboflavin status of 60 malnourished children in relation to thyroid hormones and urinary excretion of organic acids. The researchers found that although plasma FAD and FMN did not indicate global riboflavin deficiency, the synthesis of mitochondrial riboflavin cofactors was reduced in children with SAM compared with control participants.^173^ They explained this by attributing low levels of the thyroid hormone T3 in children with malnutrition as a contributor to reduced enzymatic activity responsible for the conversion of riboflavin to its cofactors.^173^

Current formulations of F-75, F-100, and RUTF provide well over the AI for riboflavin, at 468%–663%, 630%–900%, and 630%–900%, respectively, of the AI, equating to an intake of 1.95–3.2 mg/d (Figure 1, see Tables S4–S12 in the Supporting Information online). No UL has been established for riboflavin; its absorption is reported to be saturated in adults at doses exceeding 27 mg, and excess amounts are excreted in the urine.^31^ Current available data are not conclusive to suggest that a change in riboflavin supplementation for children with SAM is warranted. See Figure 18 for riboflavin take-home messages.

### Niacin

**Figure 19:**
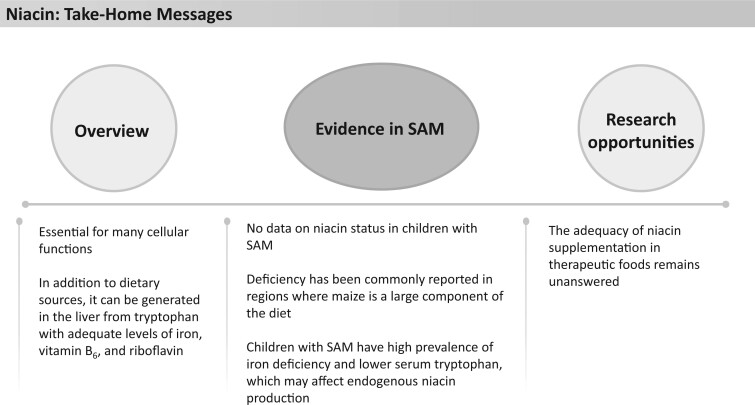
**Niacin take-home messages.** *Abbreviation:* SAM, severe acute malnutrition.

Niacin (vitamin B_3_) is an essential nutrient involved in many cellular functions, including oxidative stress regulation, mitochondrial homeostasis, autophagy, and gene expression regulation (Figure 19).^174–176^ In addition to dietary sources, niacin can also be generated in the liver from the amino acid tryptophan,^31^^,^^176^^,^^177^ which requires adequate levels of iron, vitamin B_6_, and riboflavin.^31^^,^  ^178^^,^^179^ Severe deficiency of niacin can lead to pellagra, which presents with dermatitis, dementia, diarrhea, and ultimately, death.^31^^,^^123^^,^^180^ Niacin status is ideally measured through urinary excretion of niacin metabolism products N^1^-methylnicotinamide and 2-pyridone, with a combined excretion of less than 1.5 mg/24 hours indicative of severe deficiency.^123^ Excess niacin intake, in the form of nicotinic acid (>1500 mg/d in adults) or nicotinamide (>3000 mg/d in adults), can lead to flushing, gastrointestinal upset, hepatotoxicity, and glucose intolerance. These levels are far in excess of the UL for adults (35 mg/d), but case reports and clinical trials have reported flushing effects at doses of 30–1000 mg/d resulting in withdrawal from treatment.^31^^,^^123^

There currently are no direct data on niacin status in children with SAM. However, niacin deficiency has been commonly reported in regions where maize is a large component of the diet^180^^,^^181^ and is thought to reduce niacin’s absorption.^182^ A prospective analysis of food and nutrient intakes by 1651 children at risk of nutritional edema in Malawi showed that although there was a substantially deficient intake of niacin in all children, there was no difference in intake between those who developed nutritional edema and those who did not.^183^ Children with SAM have a high prevalence of iron deficiency,^77^^,^^78^ which may affect endogenous niacin production. Furthermore, children with complicated SAM have lower serum tryptophan levels than children with stunting and control participants without stunting,^184^ which also could affect endogenous niacin production.

Current formulations of therapeutic foods contain between 111% and 237% of the AI or RDA for niacin, with F-100 and RUTF just reaching the UL for children older than 1 year and exceeding it for those older than 2 years (Figure 1, see Tables S4–S12 in the Supporting Information online). In absolute terms, this provides 3.2–14.3 mg/d (UL 10 mg/d). Niacin doses recommended for pellagra treatment in children and adolescents are 50–300 mg/d.^185^ More data on niacin status in children with SAM are required to make recommendations on niacin content in therapeutic foods. See Figure 19 for niacin take-home messages.

### Pantothenic acid

**Figure 20:**
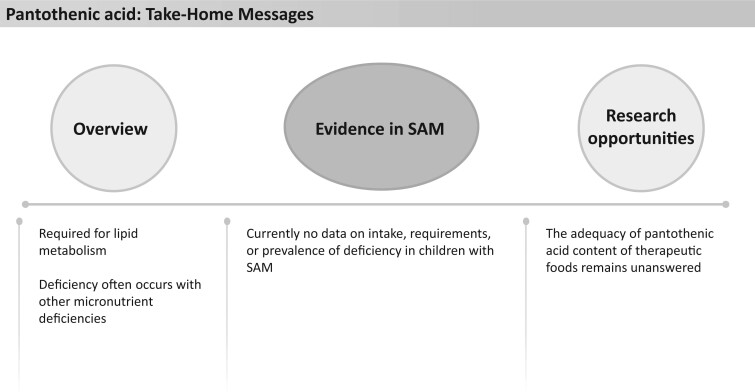
**Pantothenic acid take-home messages.** *Abbreviation:* SAM, severe acute malnutrition.

Pantothenic acid (vitamin B_5_) is an essential micronutrient needed for the synthesis of coenzyme A and acyl carrier protein, which are required for the metabolism of lipids (Figure 20).^186^^,^^187^ Deficiency of pantothenic acid often occurs in conjunction with other micronutrient deficiencies, making it difficult to determine clinical signs of deficiency.^188^ Signs of deficiency may include numbness and burning of the hands and feet, headache, fatigue, irritability, restlessness, poor sleep, and gastrointestinal disturbances.^31^ Pantothenic acid status can be measured in blood, urine, or tissue samples, but urinary excretion is most closely related to dietary intake.^31^

There currently appear to be no data on intake, requirements, or prevalence of deficiency of pantothenic acid in children with SAM. Current therapeutic foods contain between 184% and 427% of the AI for children (Figure 1, see Tables S4–S12 in the Supporting Information online). No UL for pantothenic acids is reported. There is no evidence on pantothenic acid status in children with SAM to assess the appropriateness of this amount. Studies are warranted to determine the prevalence of deficiency and establish the optimal supplementation of pantothenic acid in this population. See Figure 20 for pantothenic acid take-home messages.

### Vitamin B_6_

**Figure 21:**
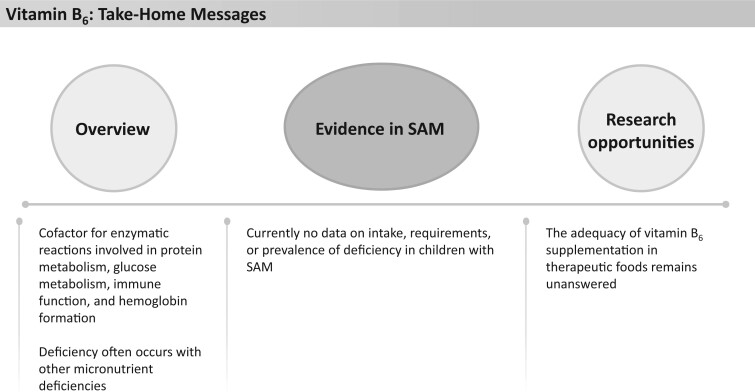
**Vitamin B_6_ take-home messages.** *Abbreviation:* SAM, severe acute malnutrition.

Vitamin B_6_, also known as pyridoxine, acts as a cofactor for more than 100 enzymatic reactions, most of which are involved with protein metabolism, including tryptophan metabolism.^27^^,^^189^ Vitamin B_6_ also plays a role in cognitive development, glucose metabolism, immune function, and hemoglobin formation (Figure 21).^27^ Isolated vitamin B_6_ deficiency is rare but may present in conjunction with other micronutrient deficiencies and is associated with microcytic anemia, dermatitis, glossitis, weakened immune function, and neurological changes.^31^ Vitamin B_6_ status can be determined by measuring concentrations of pyridoxal 5′-phosphate (a B_6_ active coenzyme form); total vitamin B_6_ in plasma, erythrocytes, or urine; or by measuring tryptophan metabolites.^31^

We found no recent data on vitamin B_6_ status in children with SAM. One study published in 1961 showed that in a small sample of children with nutritional edema (n = 13), all had evidence of B_6_ deficiency by measure of urinary tryptophan metabolites.^190^

Current therapeutic foods provide between 156% and 330% of the RDA for vitamin B_6_, which is equivalent to 0.65–1.65 mg/d (Figure 1, see Tables S4–S12 in the Supporting Information online). Treatment guidelines for vitamin B_6_ deficiency in children and adolescents suggest doses between 5 and 25 mg/d for 3 weeks, followed by 2.5–5 mg/d.^191^ Research is needed to ascertain the prevalence of vitamin B_6_ deficiency in children with SAM and to determine whether current micronutrient supplementation in therapeutic foods is optimal. See Figure 21 for vitamin B_6_ take-home messages.

### Folic acid

**Figure 22:**
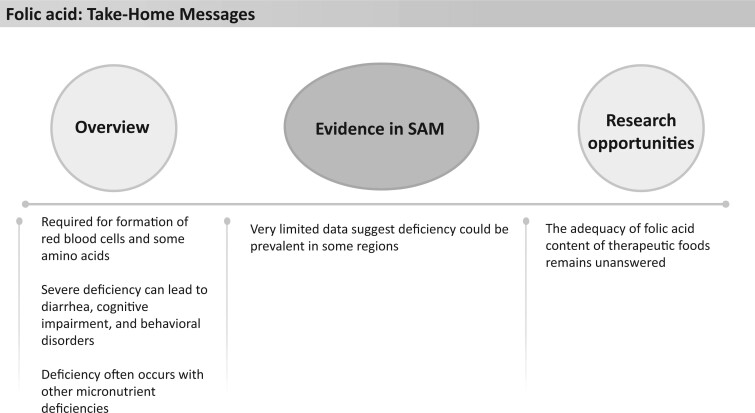
**Folic acid take-home messages.** *Abbreviation:* SAM, severe acute malnutrition.

Folic acid, or folate, is important for the formation of red blood cells and certain amino acids, especially methionine. Folic acid deficiency can present clinically with weight loss, growth delay, and megaloblastic anemia.^31^ Severe folate deficiency can also result in diarrhea, cognitive impairment, and behavioral disorders (Figure 22).^31^ Isolated folate deficiency is uncommon; it often coexists with other micronutrient deficiencies. Folic acid status is typically measured using serum folate, with less than 3 ng/mL considered deficient. However, serum folate is reflective of recent dietary intake, whereas erythrocyte folate concentrations are considered a better marker of longer-term folate status. An erythrocyte folate concentration less than 140 ng/mL is considered deficient.^31^

As with many other micronutrients, data on folic acid status in children with SAM are based on small studies, with only a few studies having been published recently. Prevalence of deficiency has been reported to be between 0% and 70%,^79^^,^^80^^,^^172^^,^^192^^,^^193^ with the majority of reports ranging between 3% and 20%.^79^^,^^80^^,^^193^ The high variability in prevalence rates has been attributed to geographic variability in dietary intake or supplemental folic acid provided to pregnant and lactating women.^80^^,^^172^^,^^193^ High rates of breastfeeding have been associated with low prevalence of folate deficiency. Folate levels in breast milk are known to be consistent regardless of maternal stores,^194^^,^^195^ which may contribute to lower rates of folate deficiency. One small study of children with SAM (n = 48) in South Africa noted a high prevalence (67%) of biochemical folate deficiency (measured by serum folate) among those patients who died (n = 6) compared with a lower prevalence of deficiency (14%) among those who survived.^196^ Additionally, a correlation with supplemental folic acid (dose not specified) and recovery from SAM was noted among 253 children with complicated SAM in Ethiopia.^197^

The previous 2003 WHO guidelines recommended 5 mg of supplemental folic acid on day 1 of treatment for complicated SAM and 1 mg/d thereafter in addition to the therapeutic feedings.^16^ However, the most recent 2013 WHO guidelines do not recommend any supplementary folic acid to what is currently in therapeutic foods,^15^ which provide between 221 and 570 µg/d folic acid (Figure 1, see Tables S4–S12 in the Supporting Information online). These estimated intakes for both F-100 and RUTF are above the UL set for children older than 12 months (300 µg/d). Given the high rates of deficiency noted in children with SAM, these high doses may be warranted. See Figure 22 for folic acid take-home messages.

### Vitamin B_12_

**Figure 23:**
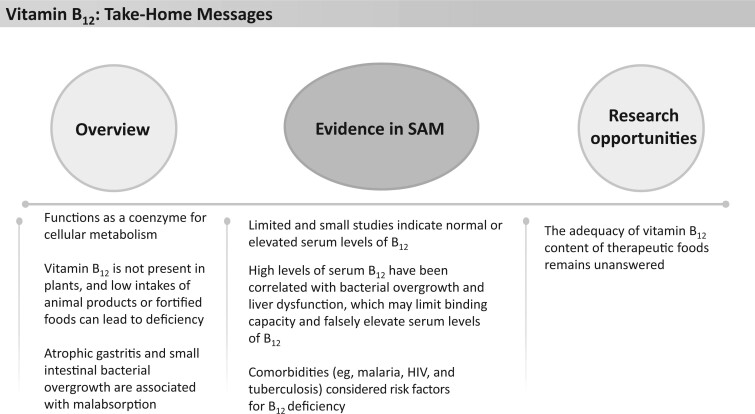
**Vitamin B_12_ take-home messages.** *Abbreviations:* HIV, human immunodeficiency virus; SAM, severe acute malnutrition.

Vitamin B_12_ (cobalamin) functions as a coenzyme important for cellular metabolism, especially for DNA synthesis, energy metabolism, and conversion of homocysteine to methionine.^198^ In children, B_12_ deficiency can lead to anemia, neurological complications such as impaired motor and cognitive functions, as well as delays in growth and neurodevelopment (Figure 23).^31^ Vitamin B_12_ status is typically measured using serum B_12_, with levels less than 148 pmol/L considered tdeficient.^199^ However more sensitive markers of B_12_ status include serum methylmalonic acid and homocysteine levels.^31^

B_12_ deficiency can occur for a number of reasons. B_12_ is not present in plants; therefore, low intake of animal products or fortified foods can lead to B_12_ deficiency.^31^ Gastrointestinal factors also play a crucial role in determining B_12_ status. The absorption of B_12_ from food is a complex process that requires the proper production of intrinsic factor, stomach acid, and functioning of the gut. Gastrointestinal conditions, such as atrophic gastritis, *Helicobacter pylori* infection, and small-intestine bacterial overgrowth, are associated with malabsorption of B_12_.^200–202^

Two small studies (n = 50 and n = 48) in India reported a high prevalence (range, 34%–58%) of vitamin B_12_ deficiency in children with SAM.^80^^,^^203^ However, the findings in these reports of relatively high prevalence of B_12_ deficiency are in contrast to other studies. A larger study in India (n = 131) showed that although 30.5% of children with SAM had megaloblastic anemia, only 6.1% had deficient serum B_12_ levels.^79^ Other studies have shown normal or elevated serum B_12_ levels in children with SAM.^193^^,^^204^^,^^205^ One of these older studies, from 1983,^205^ (n = 34) also noted that patients with nutritional edema had higher serum B_12_ levels than did children with severe wasting.^205^ It has been suggested that higher or normal levels of serum B_12_ in malnourished children do not necessarily indicate adequacy, but rather that binding capacity of B_12_ in the liver is limited due to fatty infiltrates, particularly in nutritional edema, leading to higher serum levels. Some authors also correlated high serum B_12_ levels with decreased liver function^205^ and with bacterial overgrowth in the gut.^206^ Children with SAM have evidence of bacterial overgrowth, which may be influencing serum B_12_ measurements.^207–210^ Given the reliance on plant-based foods in diets in most LMICs, it is not surprising that children with SAM would be at risk of B_12_ deficiency. Moreover, comorbidities prevalent in SAM, like malaria, human immunodeficiency virus, and tuberculosis, have also been implicated as risk factors for B_12_ deficiency.^211^^,^^212^

Most treatment guidelines suggest using intramuscular B_12_ injections to treat severe deficiencies, which is not always a feasible solution for LMICs. Current therapeutic foods provide between 260% and 525% of the RDA for vitamin B_12_, which translates to a dose of 1.95–4.5 µg/d (Figure 1, see Tables S4–S12 in the Supporting Information online). Although these doses may exceed the RDA, there is no UL reported for vitamin B_12_, and recent evidence suggests that much higher doses (1000 µg) of oral vitamin B_12_ taken for 4 months may be required to address diet-related deficiencies in children under 2 years of age.^213^ Given the low risk associated with high doses of vitamin B_12_ and that a UL has not been set for vitamin B_12_, it may be valuable to increase the amount provided in therapeutic foods. However, research with a more sensitive marker of B_12_ status, such as serum methylmalonic acid, may be required to better understand prevalence of deficiency. See Figure 23 for vitamin B_12_ take-home messages.

### Biotin

**Figure 24:**
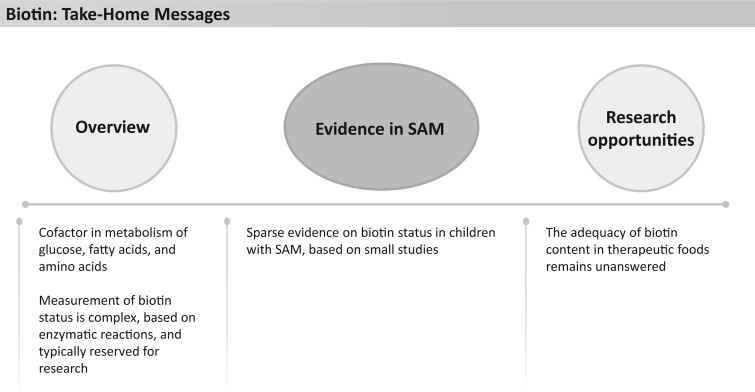
**Biotin take-home messages.** *Abbreviation:* SAM, severe acute malnutrition.

Biotin is absorbed in the small intestine and serves as a cofactor for different carboxylases, which play an essential role in the metabolism of glucose, fatty acids, and amino acids (Figure 24).^31^^,^^214^ Additionally, it is an important component of gene regulation and cell signaling.^31^^,^^215^ Clinical manifestations of deficiency are nonspecific and include periorificial dermatitis, hair loss, neurological dysfunction, and metabolic abnormalities.^31^^,^^216^ Indicators of biotin deficiency include low serum and low urinary levels of biotin, high urinary excretion of 3-hydroxyisovaleric acid in relation to creatinine excretion, biotinylated methylcrotonyl-coenzyme A carboxylase (MCC) and propionyl-coenzyme A carboxylase (PCC) in lymphocytes.^31^^,^^217^ The latter 2 are considered the most reliable indicators because serum and urinary biotin measures do not reflect marginal deficiency.^31^

Two small studies conducted by the same group in Mexico reported evidence of biotin deficiency in children with SAM on admission. In 1 study, plasma biotin concentrations and lymphocyte carboxylase activities (including PCC) were lower in 16 children with SAM compared with a control group. However, urinary biotin concentrations (expressed per gram of creatinine) were higher in children with SAM than in control participants. The authors suggested this may not be indicative of adequate intake of biotin but rather due to increased renal clearance or reduced creatinine excretion, both of which occur with malnutrition.^218^ The same group also noted reduced levels of lymphocyte PCC in 22 patients with SAM, whereas plasma biotin concentrations were not significantly different from those of healthy control participants.^219^ In that study,^219^ patients were randomized to receive either a high-dose biotin supplement (10 mg/d) or placebo for 15 days. Researchers found that children with low lymphocyte PCC levels were the only group to significantly respond to the supplement, confirming their deficiency on admission.^219^

The AI for biotin ranges from 6 to 8 μg/d, depending on age. There is no UL because no adverse effects have been reported, even at a high oral intake of 200 mg/d.^31^ Currently, therapeutic formulas F-75, F-100, and RUTF contain approximately 975%–1381%, 1444%–2062%, and 1470%–2100%, respectively, of the AI for biotin (Figure 1, see Tables S4–S12 in the Supporting Information online). This translates to daily doses of 65–168 µg/d; it has been recommended that clinical biotin deficiency in children be treated with doses of 5–40 mg/d.^220^ Sparse literature suggests that children with SAM may be at risk for biotin deficiency, which may warrant an increase in the amount contained in therapeutic foods. However, there is currently not enough evidence to make conclusive recommendations. See Figure 24 for biotin take-home messages.

## DISCUSSION

The introduction of therapeutic foods in the 1990s improved outcomes for children with SAM. However, mortality rates of children under 5 years of age with severe malnutrition continues to be high, especially in children with SAM^9^^,^^76^ Limited studies have investigated the nutritional needs of children with SAM, including caloric requirements,^221–223^ protein requirements,^224–226^ and requirements for some key micronutrients such as vitamin A, vitamin D, thiamine, iron, and selenium. However, overall, little high-quality research has been dedicated to assessing the status of most micronutrients in this population. This review illustrates that concentrations and deficiencies of most micronutrients either have not been previously reported or only reported in a few historical studies of small sample size, and these studies were generally not conducted in an RCT format or in a range of different geographies or subpopulations (eg, sick complicated vs well uncomplicated malnourished children). This is important because the definitions of and treatment protocols for severe malnutrition have changed over time, and research conducted on malnourished children in the 1970s or 1980s may not reflect the current landscape.

**Figure 25:**
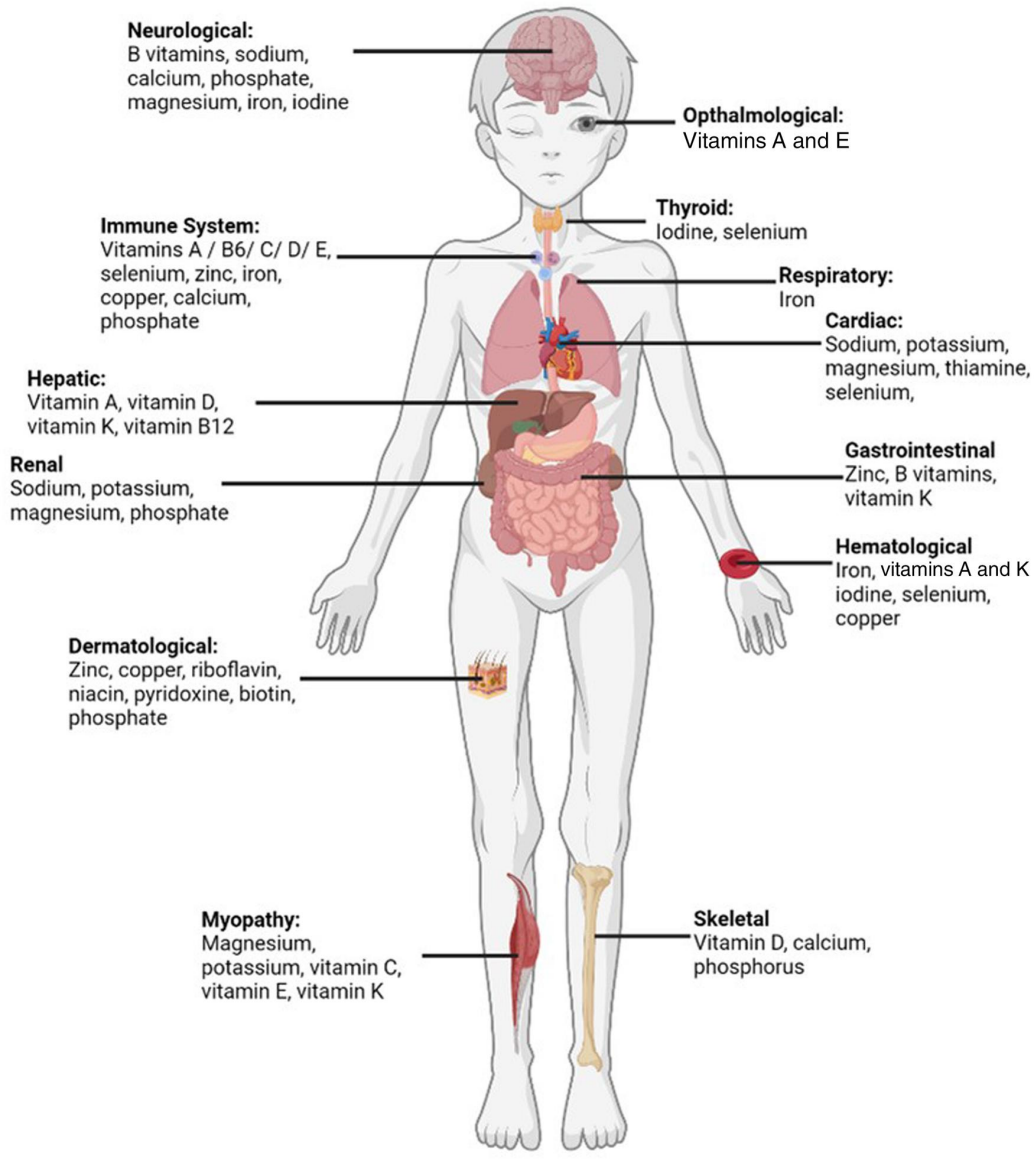
Organ systems affected by altered micronutrient levels. Created with BioRender.com.

Death remains a considerable risk for the children with SAM, even after they finish their treatment of concurrent medical conditions and nutritional rehabilitation,^11^^,^^227^ suggesting that other factors may be contributory. A 1-year follow-up study of 1024 children admitted to hospital for treatment of SAM showed that 42% of children died during or after treatment, with 25% of these deaths occurring more than 90 days after admission to hospital.^11^ A more recent and larger study of 3101 children, including 1218 children with severe malnutrition, reported an overall mortality rate of 20.4%, with 48% of patients died after initial discharge from hospital.^9^ The authors of the same study also reported on factors that were directly or indirectly associated with postdischarge death, including human immunodeficiency virus infection or exposure, underlying medical conditions, and adverse caregiver characteristics.^9^ However, given the intricate role each micronutrient has within the body, as illustrated in Figure 25, the authors believe inadequate micronutrient status may be another important contributory factor to the poor outcomes for children affected with SAM. We believe, therefore, that knowledge of micronutrient status and assessment to ensure adequate micronutrient supplementation in children with SAM should be considered a research priority to guide clinical practice.

In reviewing the literature on micronutrients, there are important relationships with organ dysfunction and micronutrient status. Although relationships of individual micronutrients to organ systems are discussed in this article, it should also be noted that micronutrients do not work in isolation; often, multiple deficiencies occur at once. From the available evidence, the combination of multiple micronutrient deficiencies is likely to have a significant impact on 1 or more systems (see Figure 25).

Micronutrient status is often different in children with nutritional edema compared with those who present with severe wasting.^83^ Children with nutritional edema may demonstrate signs of increased oxidative stress, with lower plasma levels of zinc, copper, selenium, and vitamins A and C than those with wasting, yet they tend to have higher levels of free circulating iron, more sodium and potassium pump sites per erythrocyte, and higher fat content in their livers.^83^ Hence, it is important to explore and compare the micronutrient requirements in patients with nutritional edema and patients with severe wasting.

At the time of their development in the 1990s, F-75 and F-100 were designed for inpatient treatment of both complicated and uncomplicated SAM.^14^ In 2007, United Nations agencies endorsed RUTF for the management of uncomplicated SAM in the community.^15^ From this point onward, if children did not pass an appetite test or were considered to have medical complications, they were admitted to hospital for treatment with the complicated SAM phenotype. However, the composition of foods used to treat them did not change.

Recently, specifications laid out by the joint WHO and the Food and Agriculture Organization of the United Nations “Codex” group advised on changes to the minimum and/or maximum levels of 6 micronutrients included in RUTF (potassium, calcium, phosphorous, magnesium, and vitamins A and  D), along with an increase in the essential fatty acid profile.^22^ The RUTF formulation used for comparison in this narrative review contains micronutrients that already conform to these specified changes.^18^^,^^22^

When assessing micronutrient requirements for children with severe malnutrition, it is difficult to know what constitutes adequacy or deficiency. Although in this review, we used the DRIs as a benchmark to assess micronutrient intake from therapeutic foods, these markers were developed on the basis of the needs of healthy populations and may not reflect the fundamental needs of children with severe malnutrition. It should also be noted that the average weights of children with SAM are in accordance with weights of well infants much younger than those with SAM. This may further limit the use of the DRIs, because the average weights used for their development are based on healthy, typically developing children.^23^ In our estimation, based on standard prescriptions of therapeutic foods, most of the micronutrients met or exceeded the AI or RDA for age and some exceeded the UL. The safety of high micronutrient doses, in particular those above the UL, is not well studied in the SAM population and requires close attention. Despite this, biochemical markers for some deficiencies, such as vitamin A and iron, are not always corrected at the end of therapy. This may be a result of saturation of absorption of a micronutrient due to an ongoing need for repletion or higher requirements in the setting of SAM of certain micronutrients. Nutritional therapy with some micronutrient quantities above the UL may be appropriate for children with SAM and severe micronutrient deficiencies.

Another challenge in assessing micronutrient requirements and, more importantly, in standardized micronutrient quantities in therapeutic foods, is the possible variability of micronutrient status between individual children and between different geographic districts, regions, and countries. Developing therapeutic food micronutrient formulations sufficient to replenish deficiencies or maintain adequate micronutrient levels for most children while ensuring no toxicity and reducing possible adverse effects is a challenging task. Some children or, indeed, populations in certain regions may require additional supplementation to their therapeutic foods. Large-scale studies of micronutrient status at the beginning, during, and end of SAM treatment, which cross multiple contexts or regions as well as children of varying ages, will likely help address this challenge.

It is important to realize when reviewing the available literature that micronutrient status interpretation can be difficult in any population but especially within the severely malnourished population. Multiple biochemical markers for the assessment of the same micronutrient status, different laboratory assays, and varying levels defining micronutrient deficiency complicate the interpretation of results and the comparison of study findings. Additionally, standard biochemical markers of micronutrient status can be affected by different disease states (eg, inflammation).^91^ Children with severe malnutrition have systemic inflammation.^228^ Systemic inflammation may redistribute micronutrients between tissues and fluid compartments, cause changes in production and loss of carrier proteins, and result in increased excretion of micronutrients or block their use.^229^ These processes may skew biochemical markers and affect interpretation of body stores.^91^ Although some researchers have adjusted for this inflammation when interpreting biochemical results,^230^ it is not always possible, and it remains an important element to consider in addition to recent dietary intake when assessing micronutrient status at admission.

Additionally, it is important that the bioavailability of each micronutrient in therapeutic foods be assessed. Different forms of each micronutrient (eg, heme vs non-heme iron, varying salt compounds), different forms of therapeutic foods (eg, dairy- and nondairy-based RUTF), disease states commonly seen in severely malnourished children (eg, inflammation, diarrhea, liver dysfunction), and the possibility that the interplay of micronutrients, therapeutic foods, and local diets may all interfere (or enhance, inhibit, or compete) with the bioavailability of each micronutrient.

### Future directions

Having knowledge of the micronutrient status at diagnosis, during, and at completion of treatment of SAM is important, and ensuring adequate micronutrient levels may affect the morbidity and mortality outcomes of children with SAM. Research on micronutrient status at all these points should be considered a priority in children with both complicated and uncomplicated SAM and in children of different age groups to ensure appropriate treatment is provided to address deficiencies. In addition, ideally, region- or context-specific data on micronutrient status will become available, because likely deficiencies of specific micronutrients differ between children with SAM in different regions for reasons related to underlying drivers including availability of different types of foods, infection patterns, rates of premature births and births of small-for-gestational-age babies, maternal nutritional deficits, and so forth. Although, overall, evidence on micronutrient status in children with SAM and subsequent responses to current treatment practices is limited, we recommend prioritizing the areas discussed in the following paragraphs.

Evidence reviewed strongly suggests that the amount of thiamine in therapeutic foods needs urgent attention. As noted earlier, the clinical presentation of children receiving inpatient treatment for SAM has vastly changed since the introduction of therapeutic foods, yet the level of thiamine has not. Given the low risk for toxicity and clear clinical benefit, we think it would be reasonable to increase thiamine supplementation to meet minimal recommendations for refeeding. Specifically, we concur with the suggestion of Hiffler et al^164^ that thiamine be increased in F-75 to 7.5 mg/100 mL, which would provide an estimated dose of 50–80 mg/d. Additionally, F-100 and RUTF could be increased to 1 mg/100 mL and 4.5 mg/92 g, respectively, to provide the recommended 1–2 mg/kg/d for children.^164^^,^^166^^,^^168^

As noted earlier, IDA remains prevalent among patients upon treatment completion. Promising research has shown improved rates of IDA using higher doses of iron in plant-based versions of RUTF, but the same products resulted in significantly less weight gain than traditional RUTF. It is worth noting that body composition was not commented on in this study, and slower rates of weight gain in the setting of improved body composition may be preferable.^231^ Regardless, the high prevalence of IDA at completion of treatment makes reviewing iron in therapeutic foods a priority. F-100 does not meet the RDA for iron, and although this product is typically given for a short time, it is important to note that some patients with swallowing dysfunction, such as children with cerebral palsy, rely solely on this product for their rehabilitation. We recommend that the iron content in F-100 be reevaluated and that further studies examining higher iron content in F-100 and RUTF, iron therapy beyond the duration of standard nutritional therapy, or, indeed, other forms of iron supplementation be conducted to determine the optimal iron dose for children with SAM. These studies must balance the benefits of iron repletion with the potential toxic effects of excess iron in the body, especially in sick infants and children, and the risk of altering the microbiome and stimulating replication of intestinal pathogens.

A recent pilot study has shown that high doses of vitamin D during rehabilitation have promising effects on improving developmental outcomes in children with uncomplicated SAM. With a larger RCT currently underway, it will be important to follow-up these results and consider increasing levels of vitamin D in F-100 and RUTF if this effect persists in this larger study.

## CONCLUSION

This review clearly shows there are gaps in research related to micronutrient status and requirements in children with severe wasting and nutritional edema. Moving forward, researchers should prioritize assessment of micronutrient status upon admission, during, and at completion of standard SAM treatment. Specifically, the micronutrients thiamine, iron, and vitamin D should be addressed first. Following this, the effects of either universal changes to supplementation of micronutrients in therapeutic foods or targeted additional individual micronutrient supplementations in specific populations of children with SAM can be assessed in studies. From there, meaningful changes to therapeutic foods can be assessed in studies to help decision-makers formulate policy and ultimately improve clinical practice.

## Supplementary Material

nuad165_Supplementary_Data

## Supporting Information

The following Supporting Information is available through the online version of this article at the publisher’s website.

*Table S1* **Micronutrient composition of F-75, F-100, and RUTF**

*Table S2* **Comparison of micronutrient composition of Nutriset formulated RUTF to Codex guidelines 2022**

*Table S3* **Dietary reference intake terms and definitions**

*Table S4* **Predicted intakes of micronutrients from F-75 for children 6-12 months of age weighing 5 kg**

*Table S5* **Predicted intakes of micronutrients from F-75 for children 12-23 months of age weighing 6 kg**

*Table S6* **Predicted intakes of micronutrients from F-75 for children 24–59 months of age weighing 8.5 kg**

*Table S7* **Predicted intakes of micronutrients from F-100 for children 6-12 months of age weighing 5 kg.**

*Table S8* **Predicted intakes of micronutrients from F-100 for children 12–23 months of age weighing 6 kg.**

*Table S9* **Predicted intakes of micronutrients from F-100 for children 24–59 months of age weighing 8.5 kg.**

*Table S10* **Predicted intakes of micronutrients from RUTF for children 6–12 months of age weighing 5 kg.**

*Table S11* **Predicted intakes of micronutrients from RUTF for children 12-23 months of age weighing 6 kg.**

*Table S12* **Predicted intakes of micronutrients from RUTF for children 24-59 months of age weighing 8.5 kg.**

## References

[1] Black RE , Victora CG, Walker SP, et al; Maternal and Child Nutrition Study Group. Maternal and child undernutrition and overweight in low-income and middle-income countries. Lancet. 2013;382:427–451. doi:10.1016/S0140-6736(13)60937-X 23746772

[2] Kerac M , McGrath M, Connell N, et al “Severe malnutrition”: thinking deeply, communicating simply. BMJ Glob Health. 2020;5:1–4. doi:10.1136/bmjgh-2020-003023 PMC7677332 33208313

[3] World Health Organization. WHO Child Growth Standards and the Identification of Severe Acute Malnutrition in Infants and Children. 2009. Available at: https://www.who.int/publications/i/item/9789241598163. Accessed January 18, 2024. 24809116

[4] Campion-Smith TJ , Kerac M, McGrath M, et al Antimicrobial and micronutrient interventions for the management of infants under 6 months of age identified with severe malnutrition: a literature review. PeerJ 2020;8:e9175. doi:10.7717/peerj.9175 32974089 PMC7487149

[5] UNICEF, World Health Organization, World Bank. Levels and trends in child malnutrition; UNICEF/WHO/World Bank Group joint child malnutrition estimates 2023 edition. 2023. Available at: https://data.unicef.org/resources/jme-report-2023/. Accessed January 18, 2024.

[6] Karlsson O , Kim R, Guerrero S, et al Child wasting before and after age two years: a cross-sectional study of 94 countries. EClinicalMedicine. 2022;46:101353. doi:10.1016/j.eclinm.2022.101353 35360149 PMC8961190

[7] Alvarez JL , Dent N, Browne L, et al Putting child kwashiorkor on the map. CMAM Forum Technical brief. 2016. Available at: http://s3.ennonline.net/attachments/2485/Putting-Kwashiorkor-on-the-Map.pdf. Accessed January 18, 2024.

[8] Bandsma RHJ , Voskuijl W, Chimwezi E, et al A reduced-carbohydrate and lactose-free formulation for stabilization among hospitalized children with severe acute malnutrition: a double-blind, randomized controlled trial. PLoS Med. 2019;16:e1002747. doi:10.1371/journal.pmed.1002747 30807589 PMC6390989

[9] Diallo AH , Sayeem Bin Shahid ASM, Khan AF, et al Childhood mortality during and after acute illness in Africa and South Asia: a prospective cohort study. Lancet Glob Health. 2022;10:e673–e684. doi:10.1016/S2214-109X(22)00118-8 35427524 PMC9023747

[10] Karunaratne R , Sturgeon JP, Patel R, et al Predictors of inpatient mortality among children hospitalized for severe acute malnutrition: a systematic review and meta-analysis. Am J Clin Nutr. 2020;112:1069–1079. doi:10.1093/ajcn/nqaa182 32885807 PMC7528552

[11] Kerac M , Bunn J, Chagaluka G, et al Follow-up of post-discharge growth and mortality after treatment for severe acute malnutrition (FuSAM study): a prospective cohort study. PLoS One. 2014;9:e96030. doi:10.1371/journal.pone.0096030 24892281 PMC4043484

[12] Grantham-McGregor S , Powell C, Walker S, et al The long-term follow-up of severely malnourished children who participated in an intervention program. Child Dev. 1994;65:428–439. 8013232

[13] Lelijveld N , Seal A, Wells JC, et al Chronic disease outcomes after severe acute malnutrition in Malawian children (ChroSAM): a cohort study. Lancet Glob Health. 2016;4:e654–e662. doi:10.1016/S2214-109X(16)30133-4 27470174 PMC4985564

[14] World Health Organization. *Management of Severe Malnutrition: A Manual for Physicians and Other Senior Health Workers*. 1999. Available at: https://www.who.int/publications/i/item/9241545119. Accessed January 18, 2024.

[15] World Health Organization. Guideline: updates on the management of severe acute malnutrition in infants and children. 2013. Available at: https://www.who.int/publications/i/item/9789241506328. Accessed January 18, 2024. 24649519

[16] Ashworth A , Khanum S, Jackson A, et al Guidelines for the Inpatient Treatment of Severely Malnourished Children. 2003. Available at: https://www.unscn.org/web/archives_resources/files/WHO_guidelines_for_the_inpatient_tr_223.pdf. Accessed January 18, 2024.

[17] Bhutta ZA , Berkley JA, Bandsma RHJ, et al Severe childhood malnutrition. Nat Rev Dis Primers. 2017;3:17067. doi:10.1038/nrdp.2017.67 28933421 PMC7004825

[18] Nutriset Group. Plumpy-nut. Ready-to-use therapeutic food. Available at: https://www.nutriset.fr/products/en/plumpy-nut. Accessed March 21, 2023.

[19] UNICEF Supply Division. Ready-to-use therapeutic food: market outlook. 2021. Available at: https://www.unicef.org/supply/media/7256/file/Ready-to-Use-Therapeutic-Food-Market-and-Supply-Update-March-2021.pdf. Accessed January 18, 2024.

[20] Mates E , Sadler K. Ready-to-use therapeutic food (RUTF) scoping study. 2020. Emergency Nutrition Network. Available at: https://www.ennonline.net/rutfscopingstudy. Accessed January 18, 2024.

[21] Kakietek J. Determinants of the global prices of ready-to-use therapeutic foods. Food Nutr Bull. 2018;39:435–448. doi:10.1177/0379572118767149 29732931

[22] Food and Agriculture Organization of the United Nations, World Health Organization. Guidelines for ready-to-use-therapeutic foods (RUTF). 2022. Available at: https://www.fao.org/fao-who-codexalimentarius/sh-proxy/en/%3Flnk%3D1%26url%3Dhttps%25253A%25252F%25252Fworkspace.fao.org%25252Fsites%25252Fcodex%25252FStandards%25252FCXG%252B95-2022%25252FCXG_095e.pdf&sa=U&ved=2ahUKEwiC1-Or8qb-AhXMBxAIHbtSCCUQFnoECAgQAQ&usg=AOvVaw1HtbLmPFjUP_6MDDvmo2tO#:∼:text=4.1%20Ready%2Dto%2Duse%20therapeutic,without%20medical%20complications%20with%20appetite. Accessed January 18, 2024.

[23] Institute of Medicine (US) Committee on Use of Dietary Reference Intakes in Nutrition Labeling. Dietary Reference Intakes: Guiding Principles for Nutrition Labeling and Fortification. National Academies Press; 2003. doi:10.17226/10872 24967483

[24] Institute of Medicine (US) Subcommittee on Interpretation and Uses of Dietary Reference Intakes, Institute of Medicine (US) Standing Committee on the Scientific Evaluation of Dietary Reference Intakes. DRI Dietary Reference Intakes: Applications in Dietary Assessment. National Academies Press; 2000. doi:10.17226/9956 25057725

[25] Romano C , Van Wynckel M. Recommendations for nutritional management of children with neurological impairment (NI). Espghan. 2017;65:242.

[26] Daniel AI , Bwanali M, Chimoyo Tenthani J, et al Data from the cluster-randomized controlled trial of the Kusamala Program. Available at: https://borealisdata.ca/dataset.xhtml?persistentId=doi:10.5683/SP2/F8SJBV. Accessed January 18, 2024.

[27] EFSA NDA Panel (EFSA Panel on Dietetic Products, Nutrition and Allergies). Dietary reference values for vitamin B_6_. EFSA J. 2016;14:e04485. doi:10.2903/j.efsa.2016.4485

[28] Institute of Medicine (US) Committee to Review Dietary Reference Intakes for Vitamin D and Calcium. In: Ross CA, Taylor CL, Yaktine AL, et al., eds. Dietary Reference Intakes for Calcium and Vitamin D. Washington, DC: National Academies Press; 2011. Available at: https://www.ncbi.nlm.nih.gov/books/NBK56070/. doi:10.17226/13050 21796828

[29] Institute of Medicine (US) Standing Committee on the Scientific Evaluation of Dietary Reference Intakes. Dietary Reference Intakes for Calcium, Phosphorus, Magnesium, Vitamin D, and Fluoride. National Academies Press; 1997. 23115811

[30] Institute of Medicine. Dietary Reference Intakes for Vitamin C, Vitamin E, Selenium, and Carotenoids. National Academies Press; 2000. 25077263

[31] Institute of Medicine. Dietary Reference Intakes for Thiamin, Riboflavin, Niacin, Vitamin B6, Folate, Vitamin B12, Pantothenic Acid, Biotin, and Choline. National Academies Press; 1998. doi:10.17226/6015 23193625

[32] Institute of Medicine. Dietary Reference Intakes for Vitamin A, Vitamin K, Arsenic, Boron, Chromium, Copper, Iodine, Iron, Manganese, Molybdenum, Nickel, Silicon, Vanadium, and Zinc. National Academies Press; 2001. 25057538

[33] Wen B , Brals D, Bourdon C, et al Predicting the risk of mortality during hospitalization in sick severely malnourished children using daily evaluation of key clinical warning signs. BMC Med. 2021;19:222. doi:10.1186/s12916-021-02074-6 34538239 PMC8451091

[34] Bagshaw SM , Townsend DR, McDermid RC. Disorders of sodium and water balance in hospitalized patients. Can J Anaesth. 2009;56:151–167. doi:10.1007/s12630-008-9017-2 19247764

[35] Brewster DR. Inpatient management of severe malnutrition: time for a change in protocol and practice. Ann Trop Paediatr. 2011;31:97–107. doi:10.1179/146532811X12925735813887 21575313

[36] Cloete J. Management of severe acute malnutrition. S Afr Med J. 2015;105:605–606. doi:10.7196/SAMJnew.7782 26447242

[37] Patrick J , Golden M. Leukocyte electrolytes and sodium transport in protein energy malnutrition. Am J Clin Nutr. 1977;30:1478–1481. 409272 10.1093/ajcn/30.9.1478

[38] Kumar R , Kumar P, Aneja S, et al Safety and efficacy of low-osmolarity ORS vs. modified rehydration solution for malnourished children for treatment of children with severe acute malnutrition and diarrhea: a randomized controlled trial. J Trop Pediatr. 2015;61:435–441. doi:10.1093/tropej/fmv054 26314308

[39] Kumar D , Singh B. An observational study to evaluate the spectrum of co-morbidities in severe acute malnutrition with unexpected dyselectrolytemia in diarrhea. Eur J Mol Clin Med. 2020;7:2792–2797. Available at: https://ejmcm.com/uploads/paper/475cfdff092e404ad2fa9296f9197a6f.pdf. Accessed January 18, 2024.

[40] Meena SK , Suman RL, Jain R, et al Study of serum electrolytes with different clinical co-morbidities in complicated severe acute malnutrition children aged 6 months to 5 years. Int J Contemp Pediatr. 2017;4:1426. doi:10.18203/2349-3291.ijcp20172679

[41] Raza M , Kumar S, Ejaz M, et al Electrolyte imbalance in children with severe acute malnutrition at a tertiary care hospital in Pakistan: a cross-sectional study. Cureus. 2020;12:e10541. doi:10.7759/cureus.10541 33094080 PMC7574973

[42] Rytter MJ , Babirekere-Iriso E, Namusoke H, et al Risk factors for death in children during inpatient treatment of severe acute malnutrition: a prospective cohort study. Am J Clin Nutr. 2017;105:494–502. doi:10.3945/ajcn.116.140822 28031190

[43] Zogg CK , Ahmed T, Faruque ASG, et al Predictive factors of hyponatremia in under-five severely malnourished children with pneumonia admitted to a large urban hospital in Dhaka, Bangladesh: a nested case-control design. FNS. 2013;04:398–404. doi:10.4236/fns.2013.44051

[44] Dakshayani B , Ravi M, Premalatha R. A study of serum electrolytes in severe acute malnourished children with and without complications. Indian J Child Health (Bhopal). 2018;5:120–123.

[45] Samadi AR , Wahed MA, Islam MR, et al Consequences of hyponatraemia and hypernatraemia in children with acute diarrhoea in Bangladesh. Br Med J (Clin Res Ed). 1983;286:671–673. doi:10.1136/bmj.286.6366.671 PMC1547074 6402197

[46] Shahrin L , Chisti MJ, Huq S, et al Clinical manifestations of hyponatremia and hypernatremia in under-five diarrheal children in a diarrhea hospital. J Trop Pediatr. 2016;62:206–212. doi:10.1093/tropej/fmv100 26851435

[47] Houston KA , Gibb JG, Maitland K. Oral rehydration of malnourished children with diarrhoea and dehydration: a systematic review. Wellcome Open Res. 2017;2:66–20. doi:10.12688/wellcomeopenres.12357.1 29090271 PMC5657219

[48] Houston KA , Gibb JG, Maitland K. Intravenous rehydration of malnourished children with acute gastroenteritis and severe dehydration: a systematic review. Wellcome Open Res. 2017;2:65–18. doi:10.12688/wellcomeopenres.12346.1 28944301 PMC5590082

[49] Maitland K , Olupot-Olupot P, Aloroker F, et al Gastroenteritis rehydration of children with severe acute malnutrition (GASTROSAM): a phase II randomised controlled trial: trial protocol. Wellcome Open Res. 2021;6:160. doi:10.12688/wellcomeopenres.16885.1 34286105 PMC8276193

[50] McDonough AA , Youn JH. Potassium homeostasis: the knowns, the unknowns, and the health benefits. Physiology (Bethesda). 2017;32:100–111. doi:10.1152/physiol.00022.2016 28202621 PMC5337831

[51] Pagana K , Pagana T, Pagana T. Mosby’s Diagnostic & Laboratory Test Reference. 14th ed. Elsevier; 2019.

[52] Alasad S , Salih O, Hassan M. Insight into potassium’s role in childhood mortality due to severe acute malnutrition. Sudan J Paediatr. 2019;19:44–51. doi:10.24911/SJP.106-1513711620 31384088 PMC6589795

[53] Shahid AB , Alam T, Shahrin L, et al Early management of hypokalaemia in severely malnourished children under five could help to reduce deaths in developing countries. Acta Paediatr. 2021;110:1658–1664. doi:10.1111/apa.15634 33089512

[54] Fatima B , Sheikh M, Naeem M. The serum sodium, potassium and calcium levels in children 6-59 months of age with severe acute malnutrition. Pak J Med Health Sci. 2017;11:292–294.

[55] Nichols BL , Garza C, Klish W, et al Muscle electrolytes in malnutrition syndromes of children. J Pediatr Gastroenterol Nutr. 1983;2:50–56. doi:10.1097/00005176-198302010-00006 6411889

[56] Brent B , Obonyo N, Akech S, et al Assessment of myocardial function in Kenyan children with severe, acute malnutrition. JAMA Netw Open. 2019;2:e91054. doi:10.1001/jamanetworkopen.2019.1054 PMC6583281 30901050

[57] Okinyi LK. The prevalence of refeeding syndrome among children with severe acute malnutrition: an observational study in Kenyatta National Hospital, Kenya. Med Clin Res. 2018;3:1–4.

[58] Kramer CV , Allen S. Malnutrition in developing countries. Paediatr Child Health. 2015;25:422–427.

[59] Manary MJ , Brewster DR. Potassium supplementation in Kwashiorkor. J Pediatr Gastroenterol Nutr. 1997;24:194–201. doi:10.1097/00005176-199702000-00014 9106107

[60] Chisti MJ , Salam MA, Ashraf H, et al Prevalence, clinical predictors, and outcome of hypocalcaemia in severely-malnourished under-five children admitted to an urban hospital in Bangladesh: a case-control study. J Health Popul Nutr. 2014;32:270–275. 25076664 PMC4216963

[61] Smilie C , Shah D, Batra P, et al Prevalence and predictors of hypocalcaemia in severe acute malnutrition. Public Health Nutr. 2020;23:3181–3186. doi:10.1017/S1368980020001895 32703321 PMC10200387

[62] Mishra SK, , Bastola SP, , Jha B. Biochemical nutritional indicators in children with protein energy malnutrition attending Kanti Children Hospital, Kathmandu, Nepal. Kathmandu Univ Med J. 2009;7:129–134. doi:10.3126/kumj.v7i2.2705 20071845

[63] Ngari MM , Thitiri J, Mwalekwa L, et al The impact of rickets on growth and morbidity during recovery among children with complicated severe acute malnutrition in Kenya: a cohort study. Matern Child Nutr. 2018;14: E 12569. doi:10.1111/mcn.12569 PMC5901410 29178404

[64] Caballero B , Solomons NW, Torun B, et al Calcium metabolism in children recovering from severe protein-energy malnutrition. J Pediatr Gastroenterol Nutr. 1986;5:740–745. doi:10.1097/00005176-198609000-00013 3093661

[65] Ferguson EL , Gibson RS, Thompson LU, et al Phytate, zinc, and calcium contents of 30 East African foods and their calculated phytate: Zn, Ca:phytate, and [Ca][phytate]/[Zn] molar ratios. J Food Compos Anal. 1988;1:316–325. doi:10.1016/0889-1575(88)90031-2

[66] Grenov B , Briend A, Sangild PT, et al Undernourished children and milk lactose. Food Nutr Bull. 2016;37:85–99. doi:10.1177/0379572116629024 26893059

[67] Chanchal R , Gupta S, Kanta C, et al Hypophosphataemia in severe acute malnutrition: a prospective observational study. Br J Nutr. 2019;121:306–311. doi:10.1017/S0007114518003197 30554575

[68] Hother AL , Girma T, Rytter MJH, et al Serum phosphate and magnesium in children recovering from severe acute undernutrition in Ethiopia: an observational study. BMC Pediatr. 2016;16:178. doi:10.1186/s12887-016-0712-9 27814707 PMC5097423

[69] Namusoke H , Hother AL, Rytter MJH, et al Changes in plasma phosphate during in-patient treatment of children with severe acute malnutrition: an observational study in Uganda. Am J Clin Nutr. 2016;103:551–558. doi:10.3945/ajcn.115.117374 26739034

[70] Yoshimatsu S , Hossain MI, Islam MM, et al Hypophosphatemia among severely malnourished children with sepsis in Bangladesh. Pediatr Int. 2013;55:79–84. doi:10.1111/j.1442-200X.2012.03724.x 22978457

[71] Kimutai D , Maleche-Obimbo E, Kamenwa R, et al Hypo-phosphataemia in children under five years with kwashiorkor and marasmic kwashiorkor. East Afr Med J. 2010;86:330–336. doi:10.4314/eamj.v86i7.54147 20499782

[72] Waterlow JC , Golden MH. Serum inorganic phosphate in protein-energy malnutrition. Eur J Clin Nutr. 1994;48:503–506. 7956992

[73] Jahnen-Dechent W , Ketteler M. Magnesium basics. Clin Kidney J. 2012;5: I 3–i14. doi:10.1093/ndtplus/sfr163 PMC4455825 26069819

[74] Dakshayani B , Murthy AVK, Kariyappa M. Effect of oral magnesium supplementation on serum magnesium levels in children recovering from severe acute malnutrition. Sri Lanka J Child Health. 2021;50:22.doi:10.4038/sljch.v50i1.9396

[75] World Health Organization. Guideline: daily iron supplementation in infants and children. 2016. Available at: https://www.who.int/publications/i/item/9789241549523. Accessed January 18, 2024. 27195348

[76] United Nations Children’s Fund (UNICEF). Levels and Trends in Child Malnutrition: Key Findings of the 2019 Edition of the Joint Child Malnutrition Estimates. World Health Organization, International Bank for Reconstruction and Development/The World Bank; 2019.

[77] Kangas ST , Salpéteur C, Nikièma V, et al Vitamin A and iron status of children before and after treatment of uncomplicated severe acute malnutrition. Clin Nutr. 2020;39:3512–3519. doi:10.1016/j.clnu.2020.03.016 32249112

[78] Akomo P , Bahwere P, Murakami H, et al Soya, maize and sorghum ready-to-use therapeutic foods are more effective in correcting anaemia and iron deficiency than the standard ready-to-use therapeutic food: randomized controlled trial. BMC Public Health. 2019;19:806. doi:10.1186/s12889-019-7170-x 31234806 PMC6591918

[79] Thakur N , Chandra J, Pemde H, et al Anemia in severe acute malnutrition. Nutrition. 2014;30:440–442. doi:10.1016/j.nut.2013.09.011 24332525

[80] Yaikhomba T , Poswal L, Goyal S. Assessment of iron, folate and vitamin B_12_ status in severe acute malnutrition. Indian J Pediatr. 2015;82:511–514. doi:10.1007/s12098-014-1600-7 25338494

[81] Ashour M , Salem S, El-Gadban H, et al Antioxidant status in children with protein-energy malnutrition (PEM) living in Cairo, Egypt. Eur J Clin Nutr. 1999;53:669–673. doi:10.1038/sj.ejcn.1600830 10477255

[82] Dempster WS , Sive AA, Rosseau S, et al Misplaced iron in kwashiorkor. Eur J Clin Nutr. 1995;49:208–210. 7539742

[83] Pham TPT , Alou MT, Golden MH, et al Difference between kwashiorkor and marasmus: comparative meta-analysis of pathogenic characteristics and implications for treatment. Microb Pathog. 2021;150:104702. doi:10.1016/j.micpath.2020.104702 33359074

[84] Sive AA , Dempster WS, Malan H, et al Plasma free iron: a possible cause of oedema in kwashiorkor. Arch Dis Child. 1997;76:54–56. doi:10.1136/adc.76.1.54 9059163 PMC1717041

[85] Sive AA , Dempster WS, Rosseau S, et al Bone marrow and chelatable iron in patients with protein energy malnutrition. S Afr Med J. 1996;86:1410–1413. 8980561

[86] Prentice AM , Cerami C, Mwangi MN, et al Safety of interventions to reduce nutritional anemia. In: Karakochuk CD, Zimmermann MB, Moretti D, Kraemer K (eds). Nutritional Anemia. Nutrition and Health series. Springer; 2022:281–293. doi:10.1007/978-3-031-14521-6_21

[87] Martinelli M , Strisciuglio C, Alessandrella A, et al Serum hepcidin and iron absorption in paediatric inflammatory bowel disease. J Crohns Colitis. 2016;10:566–574. doi:10.1093/ecco-jcc/jjv242 26733407 PMC4957448

[88] Drakesmith H , Prentice AM. Hepcidin and the iron-infection axis. Science. 2012;338:768–772. doi:10.1126/science.1224577 23139325

[89] Goyal A , Zheng Y, Albenberg LG, et al Anemia in children with inflammatory bowel disease: a position paper by the IBD Committee of the North American Society of Pediatric Gastroenterology, Hepatology and Nutrition. J Pediatr Gastroenterol Nutr. 2020;71:563–582. doi:10.1097/MPG.0000000000002885 32947565

[90] Mohammed NI , Wason J, Mendy T, et al A novel nano-iron supplement versus standard treatment for iron deficiency anaemia in children 6–35 months (IHAT-GUT trial): a double-blind, randomised, placebo-controlled non-inferiority phase II trial in The Gambia. EClinicalMedicine. 2023;56:101853. doi:10.1016/j.eclinm.2023.101853 36880049 PMC9985047

[91] Gerasimidis K , Bronsky J, Catchpole A, et al; ESPGHAN Committee on Nutrition. Assessment and interpretation of vitamin and trace element status in sick children: a position paper from the European Society for Paediatric Gastroenterology Hepatology, and Nutrition Committee on Nutrition. J Pediatr Gastroenterol Nutr. 2020;70:873–881. doi:10.1097/MPG.0000000000002688 32443051

[92] Bhaskaram P. Immunobiology of mild micronutrient deficiencies. Br J Nutr. 2001;85(suppl 2):S75–S80. doi:10.1079/BJN2000297 11509093

[93] Bhaskaram P , Hemalatha P. Zinc status of Indian children. Indian J Med Res. 1995;102:210–215. 8675240

[94] Caulfield L , Black R. Zinc deficiency. In: Ezzati M, Lopez A, Rodgers A, Murray C, eds. Comparative Quantification of Health Risks: Global and Regional Burden of Disease Attributable to Selected Major Risk Factors. World Health Organization; 2004:257–259.

[95] Thakur S , Gupta N, Kakkar P. Serum copper and zinc concentrations and their relation to superoxide dismutase in severe malnutrition. Eur J Pediatr. 2004;163:742–744. doi:10.1007/s00431-004-1517-7 15316774

[96] Chowdhury B , Hoque MA, Hossain MA, et al Serum zinc, copper, magnesium & phosphorus level in children with severe acute malnutrition (SAM). Mymensingh Med J. 2016;25:635–640. 27941722

[97] Ghone RA , Suryakar AN, Kulhalli PM, et al A study of oxidative stress biomarkers and effect of oral antioxidant supplementation in severe acute malnutrition. J Clin Diagn Res. 2013;7:2146–2148. published online doi:10.7860/JCDR/2013/6019.3454 24298460 PMC3842512

[98] Golden MHN , Golden BE. Effect of zinc supplementation on the dietary intake, rate of weight gain, and energy cost of tissue deposition in children recovering from severe malnutrition. Am J Clin Nutr. 1981;34:900–908. doi:10.1093/ajcn/34.5.900 6786072

[99] Makonnen B , Venter A, Joubert G. A randomized controlled study of the impact of dietary zinc supplementation in the management of children with protein–energy malnutrition in Lesotho. I: Mortality and morbidity. J Trop Pediatr. 2003;49:353–360. doi:10.1093/tropej/49.6.340 14725412

[100] Roy SK. Zinc supplementation in the treatment of childhood diarrhoea. Indian J Pediatr. 1995;62:181–193. doi:10.1007/BF02752324 10829866

[101] Sazawal S , Black RE, Bhan MK, et al Zinc supplementation in young children with acute diarrhea in India. N Engl J Med. 1995;333:839–844. doi:10.1056/NEJM199509283331304 7651474

[102] Ullah Khan W , Sellen D. Zinc supplementation in the management of diarrhoea. 2011. Available at: https://www.who.int/elena/titles/bbc/zinc_diarrhoea/. Accessed January 18, 2024.

[103] Zlotkin S. A critical assessment of the upper intake levels for infants and children. J Nutr. 2006;136:502S–506S. doi:10.1093/jn/136.2.502S 16424135

[104] Weisstaub G , Medina M, Pizarro F, et al Copper, iron, and zinc status in children with moderate and severe acute malnutrition recovered following WHO protocols. Biol Trace Elem Res. 2008;124:1. doi:10.1007/s12011-008-8090-2 18483793

[105] Doherty CP , Sarkar MA, Shakur MS, et al Zinc and rehabilitation from severe protein-energy malnutrition: higher-dose regimens are associated with increased mortality. Am J Clin Nutr. 1998;68:742–748. doi:10.1093/ajcn/68.3.742 9734756

[106] Noble CCA , Sturgeon JP, Bwakura-Dangarembizi M, et al Postdischarge interventions for children hospitalized with severe acute malnutrition: a systematic review and meta-analysis. Am J Clin Nutr. 2021;113:574–585. doi:10.1093/ajcn/nqaa359 33517377 PMC7948836

[107] Erdman JW , Macdonald IA, Zeisel SH, eds. Present Knowledge in Nutrition. Wiley-Blackwell; 2012. doi:10.1002/9781119946045

[108] Myint ZW , Oo TH, Thein KZ, et al Copper deficiency anemia: review article. Ann Hematol. 2018;97:1527–1534. doi:10.1007/s00277-018-3407-5 29959467

[109] Gautam B , Deb K, Banerjee M, et al Serum zinc and copper level in children with protein energy malnutrition. Mymensingh Med J. 2008;17(suppl 2):S12–5. 18946444

[110] Tatli MM , Vural H, Koc A, et al Altered anti-oxidant status and increased lipid peroxidation in marasmic children. Pediatr Int. 2000;42:289–292. doi:10.1046/j.1442-200x.2000.01217.x 10881588

[111] Squali Houssaïni FZ , Iraqi MR, Arnaud J, et al Trace elements and protein-calorie malnutrition in the Fès area (Morocco). Biomed Pharmacother. 1997;51:349–351. doi:10.1016/s0753-3322(97)88054-7 9436529

[112] Chuwa LM , Mwiruki G, Bilal MG, et al Serum iron, zinc, copper and bromine in malnourished children in Dar es Salaam. Tanzania. East Afr Med J. 1996;73(suppl 5):S21–S23. 8756023

[113] Singla PN , Chand P, Kumar A, et al Serum, zinc and copper levels in children with protein energy malnutrition. Indian J Pediatr. 1996;63:199–203. doi:10.1007/BF02845244 10829989

[114] Subotzky EF , Heese HD, Sive AA, et al Plasma zinc, copper, selenium, ferritin and whole blood manganese concentrations in children with kwashiorkor in the acute stage and during refeeding. Ann Trop Paediatr. 1992;12:13–22. doi:10.1080/02724936.1992.11747541 1376581

[115] Rao A , Jericho H, Patton T, et al Factors affecting selenium status in infants on parenteral nutrition therapy. J Pediatr Gastroenterol Nutr. 2021;73: E 73–e78. doi:10.1097/MPG.0000000000003174 34016878

[116] Masumoto K , Nagata K, Higashi M, et al Clinical features of selenium deficiency in infants receiving long-term nutritional support. Nutrition. 2007;23:782–787. doi:10.1016/j.nut.2007.08.001 17826957

[117] Golden MHN , Ramdath D. Free radicals in the pathogenesis of kwashiorkor. Proc Nutr Soc. 1987;46:53–68. doi:10.1079/PNS19870008 3575323

[118] Ciliberto H , Ciliberto M, Briend A, et al Antioxidant supplementation for the prevention of kwashiorkor in Malawian children: randomised, double blind, placebo controlled trial. BMJ. 2005;330:1109. doi:10.1136/bmj.38427.404259.8F 15851401 PMC557886

[119] Bebars GM , Afifi MF, Mahrous DM, et al Assessment of some micronutrients serum levels in children with severe acute malnutrition with and without cerebral palsy- a follow up case control study. Clin Nutr Exp. 2019;23:34–43. doi:10.1016/j.yclnex.2018.10.008

[120] Salih MAM , Mohamed EFH, Galgan V, et al Selenium in malnourished Sudanese children: status and interaction with clinical features. Ann Nutr Metab. 1994;38:68–74. doi:10.1159/000177795 8067687

[121] Sempértegui F , Estrella B, Vallejo W, et al Selenium serum concentrations in malnourished Ecuadorian children: a case-control study. Int J Vitam Nutr Res. 2003;73:181–186. doi:10.1024/0300-9831.73.3.181 12847994

[122] Gashu D , Stoecker BJ, Bougma K, et al Stunting, selenium deficiency and anemia are associated with poor cognitive performance in preschool children from rural Ethiopia. Nutr J. 2015;15:38. doi:10.1186/s12937-016-0155-z PMC4828825 27067274

[123] Román GC. Nutritional disorders in tropical neurology. Handb Clin Neurol. 2013;114:381–404. doi:10.1016/B978-0-444-53490-3.00030-3 23829926

[124] de Benoist B , Maria Andersson M, Egli I, Takkouche B, Allen H, eds.; World Health Organization. Iodine Status Worldwide. World Health Organization; 2004. PMC2626287 16175826

[125] Andersson M , Karumbunathan V, Zimmermann MB. Global iodine status in 2011 and trends over the past decade. J Nutr. 2012;142:744–750. doi:10.3945/jn.111.149393 22378324

[126] World Health Organization. Nutrition: effects of iodine deficiency. May 24, 2013. https://www.who.int/news-room/questions-and-answers/item/nutrition-effects-of-iodine-deficiency. Accessed December 22, 2021.

[127] Ingenbleek Y. Thyroid dysfunction in protein-calorie malnutrition. Nutr Rev. 1986;44:253–263. doi:10.1111/j.1753-4887.1986.tb07649.x 3092153

[128] Ingenbleek Y , Beckers C. Evidence for intestinal malabsorption of iodine in protein-calorie malnutrition. Am J Clin Nutr. 1973;26:1323–1330. doi:10.1093/ajcn/26.12.1323 4202248

[129] Ingenbleek Y , Beckers C. Thyroid iodide clearance and radioiodide uptake in protein-calorie malnutrition. Am J Clin Nutr. 1978;31:408–415. doi:10.1093/ajcn/31.3.408 415591

[130] Ingenbleek Y , Malvaux P. Iodine balance studies in protein-calorie malnutrition. Arch Dis Child. 1974;49:305–309. doi:10.1136/adc.49.4.305 4208456 PMC1648751

[131] Blomhoff R , Blomhoff HK. Overview of retinoid metabolism and function. J Neurobiol. 2006;66:606–630. 10.1002/neu.20242 16688755

[132] World Health Organization. Guideline: vitamin A supplementation in infants and children 6–59 months of age. 2011. Available at: https://www.who.int/publications/i/item/9789241501767. Accessed January 18, 2024. 24575452

[133] World Health Organization. Global prevalence of vitamin A deficiency in populations at risk 1995-2005. 2009. Available at: https://www.who.int/publications/i/item/9789241598019. Accessed January 18, 2024.

[134] Baeten JM , Richardson BA, Bankson DD, et al Use of serum retinol-binding protein for prediction of vitamin A deficiency: effects of HIV-1 infection, protein malnutrition, and the acute phase response. Am J Clin Nutr. 2004;79:218–225. doi:10.1093/ajcn/79.2.218 14749226

[135] Ferraz IS , Daneluzzi JC, Vannucchi H, et al Detection of vitamin A deficiency in Brazilian preschool children using the serum 30-day dose-response test. Eur J Clin Nutr. 2004;58:1372–1377. doi:10.1038/sj.ejcn.1601978 15054418

[136] Stevens GA , Bennett JE, Hennocq Q, et al Trends and mortality effects of vitamin A deficiency in children in 138 low-income and middle-income countries between 1991 and 2013: A pooled analysis of population-based surveys. Lancet Glob Health. 2015;3:e528–e536. doi:10.1016/S2214-109X(15)00039-X 26275329

[137] de Fátima Costa Caminha M , da Silva Diniz A, Falbo AR, et al Serum retinol concentrations in hospitalized severe protein-energy malnourished children. J Trop Pediatr. 2008;54:248–252. doi:10.1093/tropej/fmn018 18385151

[138] Sattar S , Ahmed T, Rasul CH, et al Efficacy of a high-dose in addition to daily low-dose vitamin a in children suffering from severe acute malnutrition with other illnesses. PLoS One. 2012;7:e33112. doi:10.1371/journal.pone.0033112 22479361 PMC3314008

[139] Roth DE , Leung M, Mesfin E, et al Vitamin D supplementation during pregnancy: state of the evidence from a systematic review of randomised trials. BMJ. 2017;359:j5237. doi:10.1136/bmj.j5237 29187358 PMC5706533

[140] Sempos CT , Binkley N. 25-Hydroxyvitamin D assay standardisation and vitamin D guidelines paralysis. Public Health Nutr. 2020;23:1153–1164. doi:10.1017/S1368980019005251 32301688 PMC7167380

[141] Roth DE , Abrams SA, Aloia J, et al Global prevalence and disease burden of vitamin D deficiency: a roadmap for action in low- and middle-income countries. Ann N Y Acad Sci. 2018;1430:44–79. doi:10.1111/nyas.13968 30225965 PMC7309365

[142] Holick MF , Binkley NC, Bischoff-Ferrari HA, et al; Endocrine Society. Evaluation, treatment, and prevention of vitamin d deficiency: an Endocrine Society clinical practice guideline. J Clin Endocrinol Metab. 2011;96:1911–1930. doi:10.1210/jc.2011-0385 21646368

[143] Vogiatzi MG , Jacobson-Dickman E, DeBoer MD; Drugs, and Therapeutics Committee of The Pediatric Endocrine Society. Vitamin D supplementation and risk of toxicity in pediatrics: a review of current literature. J Clin Endocrinol Metab. 2014;99:1132–1141. doi:10.1210/jc.2013-3655 24456284

[144] Nabeta HW , Kasolo J, Kiggundu RK, et al Serum vitamin D status in children with protein-energy malnutrition admitted to a national referral hospital in Uganda. BMC Res Notes. 2015;8:418. doi:10.1186/s13104-015-1395-2 26346815 PMC4562347

[145] Walli NZ , Munubhi EK, Aboud S, et al Vitamin D levels in malnourished children under 5 years in a tertiary care center at Muhimbili National Hospital, Dar es Salaam, Tanzania-a cross-sectional study. J Trop Pediatr. 2017;63:203–209. doi:10.1093/tropej/fmw081 27794532 PMC5914404

[146] Saleem J , Zakar R, Zakar MZ, et al High-dose vitamin D_3_ in the treatment of severe acute malnutrition: a multicenter double-blind randomized controlled trial. Am J Clin Nutr. 2018;107:725–733. doi:10.1093/ajcn/nqy027 29722846

[147] Queen Mary University of London. Trial of high-dose vitamin D in the treatment of complicated severe acute malnutrition (ViDiSAM). 2020. Available at: https://clinicaltrials.gov/ct2/show/NCT04270643. Accessed July 14, 2022.

[148] Dror DK , Allen LH. Vitamin E deficiency in developing countries. Food Nutr Bull. 2011;32:124–143. doi:10.1177/156482651103200206 22164974

[149] Field CJ , Johnson IR, Schley PD. Nutrients and their role in host resistance to infection. J Leukoc Biol. 2002;71:16–32. 11781377

[150] Abrol P , Sharma N, Lal H. Vitamin E status in protein energy malnutrition. Indian J Clin Biochem. 1997;12:125–127. doi:10.1007/BF02873675 23100878 PMC3453677

[151] Becker K , Bötticher D, Leichsenring M. Antioxidant vitamins in malnourished Nigerian children. Int J Vitam Nutr Res. 1994;64:306–310. 7883470

[152] Kalra V , Grover J, Ahuja GK, et al Vitamin E deficiency and associated neurological deficits in children with protein-energy malnutrition. J Trop Pediatr. 1998;44:291–295. doi:10.1093/tropej/44.5.291 9819493

[153] Laditan AAO , Ette SI. Plasma α-tocopherol (vitamin E) levels and tocopherol–lipid ratio among children with protein–energy malnutrition (PEM). Ann Trop Paediatr. 1982;2:85–88. doi:10.1080/02724936.1982.11748233 6185084

[154] Aziz F , Patil P. Role of prophylactic vitamin K in preventing antibiotic induced hypoprothrombinemia. Indian J Pediatr. 2015;82:363–367. doi:10.1007/s12098-014-1584-3 25297643

[155] Carr A , Maggini S. Vitamin C and immune function. Nutrients 2017;9:1211. doi:10.3390/nu9111211 29099763 PMC5707683

[156] Gershoff SN. Vitamin C (ascorbic acid): new roles, new requirements? Nutr Rev. 2009;51:313–326. doi:10.1111/j.1753-4887.1993.tb03757.x 8108031

[157] Sauberlich HE. Vitamin C status: methods and findings. Ann N Y Acad Sci. 1975;258:438–450. doi:10.1111/j.1749-6632.1975.tb29302.x 1060412

[158] Jacob RA. Assessment of human vitamin C status. J Nutr. 1990;120(suppl 11):1480–1485. doi:10.1093/jn/120.suppl_11.1480 2243292

[159] Mitmesser SH , Ye Q, Evans M, et al Determination of plasma and leukocyte vitamin C concentrations in a randomized, double-blind, placebo-controlled trial with Ester-C(^®^). Springerplus. 2016;5:1161. doi:10.1186/s40064-016-2605-7 27512620 PMC4960105

[160] Crane RJ , Jones KDJ, Berkley JA. Environmental enteric dysfunction: an overview. Food Nutr Bull. 2015;36:S76–S87. doi:10.1177/15648265150361S113 25902619 PMC4472379

[161] Akinyanju OO , Grange A, Adesemoye EF. Leucocyte ascorbic acid levels in Nigerian children with protein-energy malnutrition. Ann Trop Paediatr. 1983;3:133–135. doi:10.1080/02724936.1983.11748284 6197023

[162] Singh J , Jain D, Verma RK, et al Scurvy: a common co-morbid condition in severe acute malnutrition. Indian J Pediatr. 2015;82:761–762. doi:10.1007/s12098-014-1686-y 25655017

[163] Dhale DS, Bhongade M, Susnerwala S. Study of prevalence of various cutaneous manifestations in children suffering from severe acute malnutrition (SAM). J Med Sci Clin Res. 2016;4:12691–12698. doi:10.18535/jmscr/v4i9.63

[164] Hiffler L , Rakotoambinina B, Lafferty N, et al Thiamine deficiency in tropical pediatrics: new insights into a neglected but vital metabolic challenge. Front Nutr. 2016;3:16. doi:10.3389/fnut.2016.00016 27379239 PMC4906235

[165] Hiffler L , Adamolekun B, Fischer PR, et al Thiamine content of F-75 therapeutic milk for complicated severe acute malnutrition: time for a change? Ann N Y Acad Sci. 2017;1404:20–26. doi:10.1111/nyas.13458 28905406

[166] Cape Town Metropole Paediatric Interest Group. Refeeding syndrome: guidelines. 2009. Available at: Available at: https://16683963377019794090.googlegroups.com/attach/179aacd0bb735/Clinical20Guidelines20Refeeding20Syndrome20Paeds20Section20Only20_.pdf?part=0.1&vt=ANaJVrGwj8_ySMWkKUfmre_KA1pVNDn22sYsVPrdTX_wRRPPAobFuDeC3Kkc8IiNezJNxjbZa65ela2KjL6cYW3h6sBuBwfpD-sRU7AAkbaiRkAsKdg18i0. Accessed January 18, 2024.

[167] Pulcini CD , Zettle S, Srinath A. Refeeding syndrome. Pediatr Rev. 2016;37:516–523. doi:10.1542/pir.2015-0152 27909106

[168] Sydney Children’s Hospitals Network. Refeeding syndrome: prevention and management Policy 2022. Available at: https://www.schn.health.nsw.gov.au/_policies/pdf/2022-103.pdf. Accessed January 18, 2024.

[169] Hailemariam B , Landman JP, Jackson AA. Thiamin status in normal and malnourished children in Jamaica. Br J Nutr. 1985;53:477–483. doi:10.1079/bjn19850057 4063285

[170] Neumann CG , Swendseid ME, Jacob M, et al Biochemical evidence of thiamin deficiency in young Ghanian children. Am J Clin Nutr. 1979;32:99–104. doi:10.1093/ajcn/32.1.99 104617

[171] Becker K , Leichsenring M, Gana L, et al Glutathione and association antioxidant systems in protein energy malnutrition: results of a study in Nigeria. Free Radic Biol Med. 1995;18:257–263. doi:10.1016/0891-5849(94)e0131-2 7744309

[172] Fondu P , Hariga-Muller C, Mozes N, et al Protein-energy malnutrition and anemia in Kivu. Am J Clin Nutr. 1978;31:46–56. doi:10.1093/ajcn/31.1.46 413429

[173] Capo-Chichi CD , Feillet F, Guéant JL, et al Concentrations of riboflavin and related organic acids in children with protein-energy malnutrition. Am J Clin Nutr. 2000;71:978–986. doi:10.1093/ajcn/71.4.978 10731506

[174] Denu JM. Vitamins and aging: pathways to NAD^+^ synthesis. Cell 2007;129:453–454. doi:10.1016/j.cell.2007.04.023 17482537

[175] Fang EF , Bohr VA. NAD^+^: the convergence of DNA repair and mitophagy. Autophagy 2017;13:442–443. doi:10.1080/15548627.2016.1257467 27929719 PMC5324847

[176] Meyer-Ficca M , Kirkland JB. Niacin. Adv Nutr. 2016;7(3):556–558. doi:10.3945/an.115.011239 27184282 PMC4863271

[177] Gasperi V , Sibilano M, Savini I, et al Niacin in the central nervous system: an update of biological aspects and clinical applications. Int J Mol Sci. 2019;20(4):974. doi:10.3390/ijms20040974 PMC6412771 30813414

[178] Oduho GW , Han Y, Baker DH. Iron deficiency reduces the efficacy of tryptophan as a niacin precursor. J Nutr. 1994;124:444–450. doi:10.1093/jn/124.3.444 8120664

[179] Badawy AAB. Kynurenine pathway of tryptophan metabolism: regulatory and functional aspects. Int J Tryptophan Res. 2017;10:1178646917691938. doi:10.1177/1178646917691938 28469468 PMC5398323

[180] Weize Prinzo Z. *Pellagra and Its Prevention and Control in Major Emergencies*. World Health Organization; 2000:1–42. Available at: https://iris.who.int/bitstream/handle/10665/66704/WHO_NHD_00.10.pdf. Accessed January 18, 2024.

[181] Seal AJ , Creeke PI, Dibari F, et al Low and deficient niacin status and pellagra are endemic in postwar Angola. Am J Clin Nutr. 2007;85:218–224. doi:10.1093/ajcn/85.1.218 17209199

[182] Suri DJ , Tanumihardjo SA. Effects of different processing methods on the micronutrient and phytochemical contents of maize: from A to Z. Compr Rev Food Sci Food Saf. 2016;15:912–926. doi:10.1111/1541-4337.12216 33401800

[183] Lin CA , Boslaugh S, Ciliberto HM, et al A prospective assessment of food and nutrient intake in a population of Malawian children at risk for kwashiorkor. J Pediatr Gastroenterol Nutr. 2007;44:487–493. doi:10.1097/MPG.0b013e31802c6e57 17414147

[184] Di Giovanni V , Bourdon C, Wang DX, et al Metabolomic changes in serum of children with different clinical diagnoses of malnutrition. J Nutr. 2016;146:2436–2444. doi:10.3945/jn.116.239145 27807038 PMC5118769

[185] Roberts I. Nelson’s textbook of pediatrics (20th edn.), by R. Kliegman, B. Stanton, J. St Geme, N. Schor (eds). Pediatr Radiol. 2017;47:1364–1365. doi:10.1007/s00247-017-3907-9

[186] Bates CJ. Pantothenic Acid. In: Strain JJ, Strain, Caballero B, Sandler MJ, eds. Encyclopedia of Human Nutrition. 2nd ed. Elsevier; 2005:467–472.

[187] Tahiliani AG , Beinlich CJ, Pantothenic acid in health and disease. Vitam Horm. 1991:46:165-228. doi:10.1016/S0083-6729(08)60684-6 1746161

[188] Holt RR , Uriu-Adams JY, Keen CL. Zinc. In: Erdman JW Jr, Macdonald IA, Zeisel SH, eds. Present Knowledge in Nutrition. 10th ed. Wiley-Blackwell; 2012: 521–539. doi:10.1002/9781119946045.ch34

[189] Parra M , Stahl S, Hellmann H. Vitamin B_6_ and its role in cell metabolism and physiology. Cells 2018;7. doi:10.3390/cells7070084 PMC6071262 30037155

[190] Theron JJ , Pretorius PJ, Wolf H, et al The state of pyridoxine nutrition in patients with kwashiorkor. J Pediatr. 1961;59:439–450. doi:10.1016/S0022-3476(61)80298-9 13776249

[191] Gal P , Reed M. Medications. In: Behrman R, Kliegman R, Jenson H, eds. Nelson Textbook of Pediatrics. 18th ed. Saunders Elsevier; 2007:2955–2999.

[192] Beard JL , Gomez LH, Haas JD. Functional anemia of complicated protein-energy malnutrition at high altitude. Am J Clin Nutr. 1986;44:181–187. doi:10.1093/ajcn/44.2.181 3088972

[193] Özkale M , Sipahi T. Hematologic and bone marrow changes in children with protein-energy malnutrition. Pediatr Hematol Oncol. 2014;31:349–358. doi:10.3109/08880018.2013.813098 23987917

[194] Dror DK , Allen LH. Overview of nutrients in human milk. Adv Nutr. 2018;9:278S–294S. doi:10.1093/advances/nmy022 29846526 PMC6008960

[195] Mackey AD , Picciano MF. Maternal folate status during extended lactation and the effect of supplemental folic acid. Am J Clin Nutr. 1999;69:285–292. doi:10.1093/ajcn/69.2.285 9989694

[196] Macdougall LG , Moodley G, Eyberg C, et al Mechanisms of anemia in protein-energy malnutrition in Johannesburg. Am J Clin Nutr. 1982;35:229–235. doi:10.1093/ajcn/35.2.229 6461244

[197] Mekuria G , Derese T, Hailu G. Treatment outcome and associated factors of severe acute malnutrition among 6-59 months old children in Debre Markos and Finote Selam hospitals, Northwest Ethiopia: a retrospective cohort study. BMC Nutr. 2017;3:42. doi:10.1186/s40795-017-0161-3 32153822 PMC7050803

[198] Nielsen MJ , Rasmussen MR, Andersen CBF, et al Vitamin B_12_ transport from food to the body’s cells—a sophisticated, multistep pathway. Nat Rev Gastroenterol Hepatol. 2012;9:345–354. doi:10.1038/nrgastro.2012.76 22547309

[199] Green R , Allen LH, Bjørke-Monsen AL, et al Vitamin B_12_ deficiency. Nat Rev Dis Primers. 2017;3:17040. doi:10.1038/nrdp.2017.40 28660890

[200] Carmel R. Associations of food-cobalamin malabsorption with ethnic origin, age, *Helicobacter pylori* infection, and serum markers of gastritis. Am J Gastroenterol. 2001;96:63–70. doi:10.1016/S0002-9270(00)02246-2 11197289

[201] Sarari AS , Farraj MA, Hamoudi W, et al *Helicobacter pylori*, a causative agent of vitamin B_12_ deficiency. J Infect Dev Ctries. 2008;2:346–349. doi:10.3855/jidc.194 19745500

[202] Suter PM , Golner BB, Goldin BR, et al Reversal of protein-bound vitamin B_12_ malabsorption with antibiotics in atrophic gastritis. Gastroenterology 1991;101:1039–1045. doi:10.1016/0016-5085(91)90731-Y 1889697

[203] Vaid A , Sharma M, B J, et al Serum vitamin B_12_ levels in severe acute malnutrition hospitalized children between age group 6 months to 59 months in Kangra, India. Int J Contemp Pediatr. 2018;5:1997. doi:10.18203/2349-3291.ijcp20183546

[204] Nkrumah FK , Nathoo KJ, Sanders DM. Iron, folate and vitamin B_12_ in severe protein-energy malnutrition. Cent Afr J Med. 1988;34:39–43. 3149208

[205] Osifo OA , Laditan AA, Parmentier Y, et al Clinical significance of serum transcobalamins in protein-energy malnutrition. Clin Nutr. 1983;2:87–91. doi:10.1016/0261-5614(83)90039-0 16829416

[206] Quigley EMM , Murray JA, Pimentel M. AGA clinical practice update on small intestinal bacterial overgrowth: expert review. Gastroenterology. 2020;159:1526–1532. doi:10.1053/j.gastro.2020.06.090 32679220

[207] Million M , Diallo A, Raoult D. Gut microbiota and malnutrition. Microb Pathog. 2017;106:127–138. doi:10.1016/j.micpath.2016.02.003 26853753

[208] Million M , Tidjani Alou M, Khelaifia S, et al Increased gut redox and depletion of anaerobic and methanogenic prokaryotes in severe acute malnutrition. Sci Rep. 2016;6:26051. doi:10.1038/srep26051 27183876 PMC4869025

[209] Smith MI , Yatsunenko T, Manary MJ, et al Gut microbiomes of Malawian twin pairs discordant for kwashiorkor. Science. 2013;339:548–554. doi:10.1126/science.1229000 23363771 PMC3667500

[210] Tidjani Alou M , Million M, Traore SI, et al Gut bacteria missing in severe acute malnutrition, can we identify potential probiotics by culturomics? Front Microbiol. 2017;8:899. doi:10.3389/fmicb.2017.00899 28588566 PMC5440526

[211] James JS. Low vitamin B-12 blood levels associated with faster progression to AIDS. AIDS Treat News. 1997;264:3–4. 11364102

[212] Premkumar M , Gupta N, Singh T, et al Cobalamin and folic acid status in relation to the etiopathogenesis of pancytopenia in adults at a tertiary care centre in North India. Anemia 2012;2012:707402. doi:10.1155/2012/707402 22545211 PMC3321527

[213] Bahadir A , Reis PG, Erduran E. Oral vitamin B_12_ treatment is effective for children with nutritional vitamin B_12_ deficiency. J Paediatr Child Health. 2014;50:721–725. doi:10.1111/jpc.12652 24944005

[214] McMahon RJ. Biotin in metabolism and molecular biology. Annu Rev Nutr. 2002;22:221–239. doi:10.1146/annurev.nutr.22.121101.112819 12055344

[215] Said HM. Biotin: biochemical, physiological and clinical aspects. Subcell Biochem. 2012;56:1–19. doi:10.1007/978-94-007-2199-9_1 22116691

[216] Zempleni J , Hassan YI, Wijeratne SS. Biotin and biotinidase deficiency. Expert Rev Endocrinol Metab. 2008;3:715–724. doi:10.1586/17446651.3.6.715 19727438 PMC2726758

[217] Mock DM. Biotin: from nutrition to therapeutics. J Nutr. 2017;147:1487–1492. doi:10.3945/jn.116.238956 28701385 PMC5525106

[218] Velázquez A , Martín-del-Campo C, Báez A, et al Biotin deficiency in protein-energy malnutrition. Eur J Clin Nutr. 1989;43:169–173. 2499449

[219] Velázquez A , Terán M, Báez A, et al Biotin supplementation affects lymphocyte carboxylases and plasma biotin in severe protein-energy malnutrition. Am J Clin Nutr. 1995;61:385–391. doi:10.1093/ajcn/61.2.385 7840079

[220] Gehrig KA , Dinulos JG. Acrodermatitis due to nutritional deficiency. Curr Opin Pediatr. 2010;22:107–112. doi:10.1097/MOP.0b013e328335107f 19966568

[221] Chase RP , Kerac M, Grant A, et al Acute malnutrition recovery energy requirements based on mid-upper arm circumference: secondary analysis of feeding program data from 5 countries, Combined Protocol for Acute Malnutrition Study (ComPAS) Stage 1. PLoS One. 2020;15: E 0230452. doi:10.1371/journal.pone.0230452 PMC7269364 32492023

[222] Isanaka S , Andersen CT, Hanson KE, et al Energy needs in the treatment of uncomplicated severe acute malnutrition: secondary analysis to optimize delivery of ready-to-use therapeutic foods. Matern Child Nutr. 2020;16: E 12989. doi:10.1111/mcn.12989 PMC7507348 32144946

[223] Nikièma V , Kangas ST, Salpéteur C, et al Adequacy of nutrient intakes of severely and acutely malnourished children treated with different doses of ready-to-use therapeutic food in Burkina Faso. J Nutr. 2021;151:1008–1017. doi:10.1093/jn/nxaa393 33571369 PMC8030704

[224] Kohlmann K , Callaghan-Gillespie M, Gauglitz JM, et al Alternative ready-to-use therapeutic food yields less recovery than the standard for treating acute malnutrition in children from Ghana. Glob Health Sci Pract. 2019;7:203–214. doi:10.9745/GHSP-D-19-00004 31189698 PMC6641811

[225] Kulkarni B , Mamidi RS. Nutrition rehabilitation of children with severe acute malnutrition: revisiting studies undertaken by the National Institute of Nutrition. Indian J Med Res. 2019;150:139–152. doi:10.4103/ijmr.IJMR_1905_18 31670269 PMC6829782

[226] Manary M , Callaghan-Gillespie M. Role of optimized plant protein combinations as a low-cost alternative to dairy ingredients in foods for prevention and treatment of moderate acute malnutrition and severe acute malnutrition. Nestle Nutr Inst Workshop Ser. 2020;93:111–120. doi:10.1159/000503347 31991424

[227] Bwakura-Dangarembizi M , Dumbura C, Amadi B, et al; The HOPE-SAM Study Team. Risk factors for post discharge mortality following hospitalization for severe acute malnutrition in Zimbabwe and Zambia. Am J Clin Nutr. 2021;113:665–674. doi:10.1093/ajcn/nqaa346 33471057 PMC7948837

[228] Attia S , Versloot CJ, Voskuijl W, et al Mortality in children with complicated severe acute malnutrition is related to intestinal and systemic inflammation: an observational cohort study. Am J Clin Nutr. 2016;104:1441–1449. doi:10.3945/ajcn.116.130518 27655441 PMC5081715

[229] Shenkin A. The key role of micronutrients. Clin Nutr. 2006;25:1–13. doi:10.1016/j.clnu.2005.11.006 16376462

[230] Namaste SM , Ou J, Williams AM, et al Adjusting iron and vitamin A status in settings of inflammation: a sensitivity analysis of the Biomarkers Reflecting Inflammation and Nutritional Determinants of Anemia (BRINDA) approach. Am J Clin Nutr. 2020;112:458S–467S. doi:10.1093/ajcn/nqaa141 PMC7396268 32743650

[231] Kangas ST , Kaestel P, Salpéteur C, et al Body composition during outpatient treatment of severe acute malnutrition: results from a randomised trial testing different doses of ready-to-use therapeutic foods. Clin Nutr. 2020;39:3426–3433. doi:10.1016/j.clnu.2020.02.038 32184026 PMC11346517

