# Supplementary material for: Micronutrient status in children aged 6–59 months with severe wasting and/or nutritional edema: implications for nutritional rehabilitation formulations
Source: Nutr Rev. 2024 Feb 13;83(1):112–45. doi: 10.1093/nutrit/nuad165 (PMC11632376; doi:10.1093/nutrit/nuad165)
Supplement: nuad165_Supplementary_Data [file nuad165_supplementary_data.zip › nuad165_Supplementary_Data/Publication License Nov-27-2023 (2).pdf]

49 Spadina Ave. Suite 200
Toronto ON M5V 2J1 Canada
www.biorender.com

Confirmation of Publication and Licensing Rights

November 27th, 2023
Science Suite Inc.

Subscription: *Student Plan*
Agreement number: *JA265DC8E4*
Journal name: *Nutrition Reviews*

To whom this may concern,

This document is to confirm that Mary Flanagan has been granted a license to use the BioRender content, including icons, templates and other original artwork, appearing in the attached completed graphic pursuant to BioRender's Academic License Terms. This license permits BioRender content to be sublicensed for use in journal publications.

All rights and ownership of BioRender content are reserved by BioRender. All completed graphics must be accompanied by the following citation: “Created with BioRender.com”.

BioRender content included in the completed graphic is not licensed for any commercial uses beyond publication in a journal. For any commercial use of this figure, users may, if allowed, recreate it in BioRender under an Industry BioRender Plan.

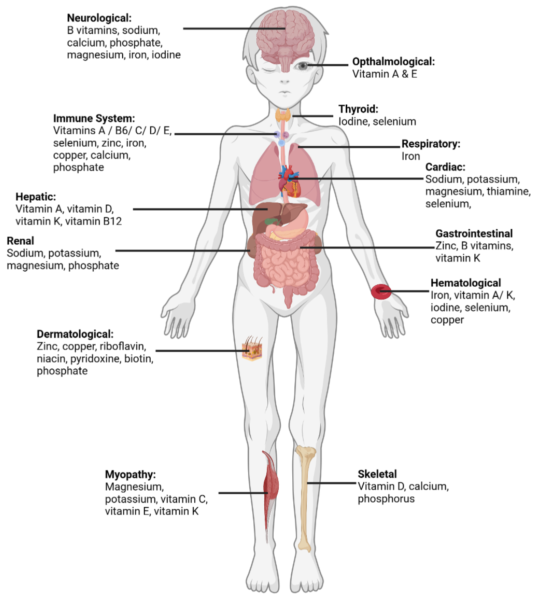

For any questions regarding this document, or other questions about publishing with BioRender refer to our BioRender Publication Guide, or contact BioRender Support at support@biorender.com.
