# Supplementary material for: Micronutrient status in children aged 6–59 months with severe wasting and/or nutritional edema: implications for nutritional rehabilitation formulations
Source: Nutr Rev. 2024 Feb 13;83(1):112–45. doi: 10.1093/nutrit/nuad165 (PMC11632376; doi:10.1093/nutrit/nuad165)
Supplement: nuad165_Supplementary_Data [file nuad165_supplementary_data.zip › nuad165_Supplementary_Data/Supplementary Tables.docx]

**Supplementary Table S1.** Micronutrient composition of F-75, F-100, and RUTF

| **Micronutrient** | **F-75**  **(per 100 mL)** | **F-100**  **(per 100 mL)** | **RUTF**  **(per 92 g)** |
| --- | --- | --- | --- |
| **Sodium (mg)** | < 17 | < 56 | 165 |
| **Potassium (mg)** | 142 | 228 | 1171 |
| **Calcium (mg)** | 77 | 76 | 302 |
| **Phosphorus (mg)** | 77 | 76 | 343 |
| **Magnesium (mg)** | 9.5 | 21 | 80 |
| **Iron (mg)** | < 0.06 | < 0.07 | 10.3 |
| **Zinc (mg)** | 2.5 | 2.4 | 11.8 |
| **Copper (µg)** | 270 | 300 | 1500 |
| **Selenium (µg)** | 5.1 | 5.7 | 28 |
| **Iodine (µg)** | 17.9 | 20 | 98 |
| **Vitamin A (mg)** | 0.14-0.3 | 0.15-0.3 | 0.79 |
| **Vitamin D3 (µg)** | 2.6-4.9 | 2.9-5.7 | 14 |
| **Vitamin E (mg)** | 3.4-4.9 | 3.8-5.7 | 18.4 |
| **Vitamin K (µg)** | 2.6-8.2 | 2.8-9.5 | 14.4 |
| **Ascorbic acid (mg)** | > 8.5 | 9.5 | 46 |
| **Thiamine (mg)** | > 0.085 | 0.095 | 0.46 |
| **Riboflavin (mg)** | > 0.3 | 0.3 | 1.5 |
| **Niacin (mg)** | > 0.85 | > 0.95 | 4.6 |
| **Pantothenic acid (mg)** | > 0.51 | > 0.57 | 2.8 |
| **Vitamin B6 (mg)** | > 0.1 | 0.1 | 0.55 |
| **Folic acid (µg)** | > 34 | 38 | 184 |
| **Vitamin B12 (µg)** | > 0.3 | 0.3 | 1.5 |
| **Biotin (µg)** | > 10 | 11 | 56 |

As per Nutriset® March 23

**Supplementary Table S2.** Comparison of micronutrient composition of Nutriset formulated RUTF to Codex Guidelines 2022

| **Micronutrient** | **Nutriset RUTF /500kcal*** | **Codex RUTF Min/500kcal** | **Codex RUTF Max/500kcal** |
| --- | --- | --- | --- |
| **Sodium (mg)** | 165 | ND | 280 |
| **Potassium (mg)** | 1171 | 1000 | 1540 |
| **Calcium (mg)** | 302 | 275 | 755 |
| **Phosphorus (mg)** | 343 | 275 | 755 |
| **Magnesium (mg)** | 80 | 75 | 225 |
| **Iron (mg)** | 10.3 | 9 | 13.5 |
| **Zinc (mg)** | 11.8 | 10 | 13.5 |
| **Copper (µg)** | 1500 | 1250 | 1750 |
| **Selenium (µg)** | 28 | 18 | 40 |
| **Iodine (µg)** | 98 | 65 | 135 |
| **Vitamin A (mg)** | 0.79 | 0.725 | 1.54 |
| **Vitamin D3 (µg)** | 14 | 13.5 | 21 |
| **Vitamin E (mg)** | 18.4 | 18 | ND |
| **Vitamin K (µg)** | 14.4 | 13.5 | 30 |
| **Ascorbic acid (mg)** | 46 | 45 | ND |
| **Thiamine (mg)** | 0.46 | 0.45 | ND |
| **Riboflavin (mg)** | 1.5 | 1.45 | ND |
| **Niacin (mg)** | 4.6 | 4.55 | ND |
| **Pantothenic acid (mg)** | 2.8 | 2.75 | ND |
| **Vitamin B6 (mg)** | 0.55 | 0.55 | ND |
| **Folic acid (µg)** | 184 | 180 | ND |
| **Vitamin B12 (µg)** | 1.5 | 1.45 | ND |
| **Biotin (µg)** | 56 | 55 | ND |

*****The values given in this table are based on Nutriset's knowledge of the intrinsic nutrient content of the raw materials and their variability, as well as the variability of the process.^18,22^

**Supplementary Table S3:** Dietary Reference Intake Terms and Definitions

| **Dietary Reference Intake Term** | **Definition** |
| --- | --- |
| **Recommended Daily Allowance (RDA)**  **Estimated Average Requirement (EAR)**  **Adequate Intake (AI)***  **Tolerable Upper Limit (UL)** | The average daily dietary nutrient intake level sufficient to meet the nutrient requirement of nearly all (97 to 98 percent) healthy individuals in a particular life stage and gender group  The average daily nutrient intake level estimated to meet the requirement of half the healthy individuals in a particular life stage and gender group  Intake levels or approximations of observed mean nutrient intakes by a group or groups of apparently healthy people who are maintaining a defined nutritional state or criterion of adequacy  The highest average daily nutrient intake level that is likely to pose no risk of adverse health effects to almost all individuals in the general population |

*Derived when there is insufficient evidence to establish an EAR or RDA

**Supplementary Table S4a:** Predicted intakes of micronutrients from F-75 for children 6-12 months of age weighing 5kg

| **Micronutrient** | **Amount Provided** | **RDA/AI** | **UL** | **%RDA/AI** | **%UL** |
| --- | --- | --- | --- | --- | --- |
| Sodium (mg)* | 110.5 | 370 | ND | 30 |  |
| Potassium (mg)* | 923 | 860 | ND | 107 |  |
| Calcium (mg)* | 500.5 | 260 | 1500 | 193 | 33 |
| Phosphorus (mg)* | 500.5 | 275 | ND | 182 |  |
| Magnesium (mg)* | 61.75 | 75 | ND | 82 |  |
| Iron (mg) | 0.39 | 11 | 40 | 4 | 1 |
| Zinc (mg) | 16.25 | 3 | 5 | 542 | 325 |
| Copper (µg)* | 1755 | 220 | ND | 798 |  |
| Selenium (µg)* | 33.15 | 20 | 60 | 166 | 55 |
| Iodine (µg)* | 116.35 | 130 | ND | 90 |  |
| Vitamin A (µg)* | 1430 | 500 | 600 | 286 | 238 |
| Vitamin D3 (µg)* | 24.375 | 10 | 38 | 244 | 64 |
| Vitamin E (mg)* | 26.975 | 5 | ND | 540 |  |
| Vitamin K (µg)* | 35.1 | 2.5 | ND | 1404 |  |
| Ascorbic Acid (mg)* | 55.25 | 50 | ND | 111 |  |
| Thiamine (mg)* | 0.5525 | 0.3 | ND | 184 |  |
| Riboflavine (mg)* | 1.95 | 0.4 | ND | 488 |  |
| Niacin (mg)* | 5.525 | 4 | ND | 138 |  |
| Pantothenic Acid (mg)* | 3.315 | 1.8 | ND | 184 |  |
| Vitamin B6 (mg)* | 0.65 | 0.3 | ND | 217 |  |
| Folic Acid (µg)* | 221 | 80 | ND | 276 |  |
| Vitamin B12 (µg)* | 1.95 | 0.5 | ND | 390 |  |
| Biotin (µg)* | 65 | 6 | ND | 1083 |  |

RDA – Recommended Daily Allowance; AI – Adequate Intake; UL - Tolerable Upper Limit; ND – Not Determinable; *Indicates only AI (not RDA) is available for this micronutrient for this age group

**Supplementary Table S4b:** Predicted intakes of micronutrients from F-75 for children 12-23 months of age weighing 6kg

| **Micronutrient** | **Amount Provided** | **RDA/AI** | **UL** | **%RDA/AI** | **%UL** |
| --- | --- | --- | --- | --- | --- |
| Sodium (mg)* | 132.6 | 1000 | 1500 | 13 | 9 |
| Potassium (mg)* | 1107.6 | 3000 | ND | 37 |  |
| Calcium (mg) | 600.6 | 700 | 2500 | 86 | 24 |
| Phosphorus (mg) | 600.6 | 460 | 3000 | 131 | 20 |
| Magnesium (mg) | 74.1 | 80 | 65^#^ | 93 | 114 |
| Iron (mg) | 0.468 | 7 | 40 | 7 | 1 |
| Zinc (mg) | 19.5 | 3 | 7 | 650 | 279 |
| Copper (µg) | 2106 | 340 | 1000 | 619 | 211 |
| Selenium (µg) | 39.78 | 20 | 90 | 199 | 44 |
| Iodine (µg) | 139.62 | 90 | 200 | 155 | 70 |
| Vitamin A (µg) | 1716 | 300 | 600 | 572 | 286 |
| Vitamin D3 (µg) | 29.25 | 15 | 63 | 195 | 46 |
| Vitamin E (mg) | 32.37 | 6 | 200 | 540 | 16 |
| Vitamin K (µg)* | 42.12 | 30 | ND | 140 |  |
| Ascorbic Acid (mg) | 66.3 | 15 | 400 | 442 | 17 |
| Thiamine (mg) | 0.663 | 0.5 | ND | 133 |  |
| Riboflavine (mg) | 2.34 | 0.5 | ND | 468 |  |
| Niacin (mg) | 6.63 | 6 | 10 | 111 | 66 |
| Pantothenic Acid (mg)* | 3.978 | 2 | ND | 199 |  |
| Vitamin B6 (mg) | 0.78 | 0.5 | 30 | 156 | 3 |
| Folic Acid (µg) | 265.2 | 150 | 300 | 177 | 88 |
| Vitamin B12 (µg) | 2.34 | 0.9 | ND | 260 |  |
| Biotin (µg)* | 78 | 8 | ND | 975 |  |

RDA – Recommended Daily Allowance; AI – Adequate Intake; UL - Tolerable Upper Limit; ND – Not Determinable; *Indicates only AI (not RDA) is available for this micronutrient for this age group; ^#^UL for magnesium represent intake from a pharmacological agent only and do not include intake from food and water.

**Supplementary Table S4c:** Predicted intakes of micronutrients from F-75 for children 24-59 months of age weighing 8.5kg

| **Micronutrient** | **Amount Provided** | **RDA/AI** | **UL** | **%RDA/AI** | **%UL** |
| --- | --- | --- | --- | --- | --- |
| Sodium (mg)* | 187.85 | 1000 | 1500 | 18.8 | 12.5 |
| Potassium (mg)* | 1569.1 | 3000 | ND | 52.3 | ND |
| Calcium (mg) | 850.85 | 700 | 2500 | 121.6 | 34.0 |
| Phosphorus (mg) | 850.85 | 460 | 3000 | 185.0 | 28.4 |
| Magnesium (mg) | 104.975 | 80 | 65 | 131.2 | 161.5 |
| Iron (mg) | 0.663 | 7 | 40 | 9.5 | 1.7 |
| Zinc (mg) | 27.625 | 3 | 7 | 920.8 | 394.6 |
| Copper (µg) | 2983.5 | 340 | 1000 | 877.5 | 298.4 |
| Selenium (µg) | 56.355 | 20 | 90 | 281.8 | 62.6 |
| Iodine (µg) | 197.795 | 90 | 200 | 219.8 | 98.9 |
| Vitamin A (µg) | 2431 | 300 | 600 | 810.3 | 405.2 |
| Vitamin D3 (µg) | 41.4375 | 15 | 63 | 276.3 | 65.8 |
| Vitamin E (mg) | 45.8575 | 6 | 200 | 764.3 | 22.9 |
| Vitamin K (µg)* | 59.67 | 30 | ND | 198.9 | ND |
| Ascorbic Acid (mg) | 93.925 | 15 | 400 | 626.2 | 23.5 |
| Thiamine (mg) | 0.93925 | 0.5 | ND | 187.9 | ND |
| Riboflavine (mg) | 3.315 | 0.5 | ND | 663.0 | ND |
| Niacin (mg) | 9.3925 | 6 | 10 | 156.5 | 93.9 |
| Pantothenic Acid (mg)* | 5.6355 | 2 | ND | 281.8 | ND |
| Vitamin B6 (mg) | 1.105 | 0.5 | 30 | 221.0 | 3.7 |
| Folic Acid (µg) | 375.7 | 150 | 300 | 250.5 | 125.2 |
| Vitamin B12 (µg) | 3.315 | 0.9 | ND | 368.3 | ND |
| Biotin (µg)* | 110.5 | 8 | ND | 1381.3 | ND |

RDA – Recommended Daily Allowance; AI – Adequate Intake; UL - Tolerable Upper Limit; ND – Not Determinable; *Indicates only AI (not RDA) is available for this micronutrient for this age group; ^#^UL for magnesium represent intake from a pharmacological agent only and do not include intake from food and water.

**Supplementary Table S4d:** Predicted intakes of micronutrients from F-100 for children 6-12 months of age weighing 5kg

| Micronutrient | Amount Provided | RDA/AI | UL | %RDA/AI | %UL |
| --- | --- | --- | --- | --- | --- |
| Sodium (mg)* | 490.0 | 370 | ND | 132 |  |
| Potassium (mg)* | 1995.0 | 860 | ND | 232 |  |
| Calcium (mg)* | 665.0 | 260 | 1500 | 256 | 44 |
| Phosphorus (mg)* | 665.0 | 275 | ND | 242 |  |
| Magnesium (mg)* | 183.8 | 75 | ND | 245 |  |
| Iron (mg) | 0.6 | 11 | 40 | 6 | 2 |
| Zinc (mg) | 21.0 | 3 | 5 | 700 | 420 |
| Copper (µg)* | 2625.0 | 220 | ND | 119 |  |
| Selenium (µg)* | 49.9 | 20 | 60 | 249 | 83 |
| Iodine (µg)* | 175.0 | 130 | ND | 135 |  |
| Vitamin A (µg)* | 1968.8 | 500 | 600 | 394 | 328 |
| Vitamin D3 (µg)* | 37.6 | 10 | 38 | 376 | 99 |
| Vitamin E (mg)* | 41.6 | 5 | ND | 831 |  |
| Vitamin K (µg)* | 53.8 | 2.5 | ND | 2153 |  |
| Ascorbic Acid (mg)* | 83.1 | 50 | ND | 166 |  |
| Thiamine (mg)* | 0.1 | 0.3 | ND | 32 |  |
| Riboflavine (mg)* | 2.6 | 0.4 | ND | 656 |  |
| Niacin (mg)* | 8.3 | 4 | ND | 208 |  |
| Pantothenic Acid (mg)* | 5.0 | 1.8 | ND | 277 |  |
| Vitamin B6 (mg)* | 0.9 | 0.3 | ND | 292 |  |
| Folic Acid (µg)* | 332.5 | 80 | ND | 416 |  |
| Vitamin B12 (µg)* | 2.6 | 0.5 | ND | 520 |  |
| Biotin (µg)* | 96.3 | 6 | ND | 1604 |  |

RDA – Recommended Daily Allowance; AI – Adequate Intake; UL - Tolerable Upper Limit; ND – Not Determinable; *Indicates only AI (not RDA) is available for this micronutrient for this age group

**Supplementary Table S4e:** Predicted intakes of micronutrients from F-100 for children 12-23 months of age weighing 6kg

| **Micronutrient** | **Amount Provided** | **RDA/AI** | **UL** | **%RDA/AI** | **%UL** |
| --- | --- | --- | --- | --- | --- |
| Sodium (mg)* | 588.0 | 1000 | 1500 | 59 | 39 |
| Potassium (mg)* | 2394.0 | 3000 | ND | 80 |  |
| Calcium (mg) | 798.0 | 700 | 2500 | 114 | 32 |
| Phosphorus (mg) | 798.0 | 460 | 3000 | 174 | 27 |
| Magnesium (mg) | 220.5 | 80 | 65^#^ | 276 | 339 |
| Iron (mg) | 0.7 | 7 | 40 | 11 | 2 |
| Zinc (mg) | 25.2 | 3 | 7 | 840 | 360 |
| Copper (µg) | 3150.0 | 340 | 1000 | 927 | 315 |
| Selenium (µg) | 59.9 | 20 | 90 | 299 | 67 |
| Iodine (µg) | 210.0 | 90 | 200 | 233 | 105 |
| Vitamin A (µg) | 2362.5 | 300 | 600 | 788 | 394 |
| Vitamin D3 (µg) | 45.2 | 15 | 63 | 301 | 72 |
| Vitamin E (mg) | 49.9 | 6 | 200 | 831 | 25 |
| Vitamin K (µg)* | 64.6 | 30 | ND | 215 |  |
| Ascorbic Acid (mg) | 99.8 | 15 | 400 | 665 | 25 |
| Thiamine (mg) | 0.1 | 0.5 | ND | 19 |  |
| Riboflavine (mg) | 3.2 | 0.5 | ND | 630 |  |
| Niacin (mg) | 10.0 | 6 | 10 | 166 | 100 |
| Pantothenic Acid (mg)* | 6.0 | 2 | ND | 299 |  |
| Vitamin B6 (mg) | 1.1 | 0.5 | 30 | 210 | 4 |
| Folic Acid (µg) | 399.0 | 150 | 300 | 266 | 133 |
| Vitamin B12 (µg) | 3.2 | 0.9 | ND | 350 |  |
| Biotin (µg)* | 115.5 | 8 | ND | 1444 |  |

RDA – Recommended Daily Allowance; AI – Adequate Intake; UL - Tolerable Upper Limit; ND – Not Determinable; *Indicates only AI (not RDA) is available for this micronutrient for this age group; ^#^UL for magnesium represent intake from a pharmacological agent only and do not include intake from food and water.

**Supplementary Table S4f:** Predicted intakes of micronutrients from F-100 for children 24-59 months of age weighing 8.5kg

| **Micronutrient** | **Amount Provided** | **RDA/AI** | **UL** | **%RDA/AI** | **%UL** |
| --- | --- | --- | --- | --- | --- |
| Sodium (mg)* | 840.0 | 1000 | 1500 | 84.0 | 56.0 |
| Potassium (mg)* | 3420.0 | 3000 | ND | 114.0 |  |
| Calcium (mg) | 1140.0 | 700 | 2500 | 162.9 | 45.6 |
| Phosphorus (mg) | 1140.0 | 460 | 3000 | 247.8 | 38.0 |
| Magnesium (mg) | 315.0 | 80 | 65^#^ | 393.8 | 484.6 |
| Iron (mg) | 1.1 | 7 | 40 | 15.0 | 2.6 |
| Zinc (mg) | 36.0 | 3 | 7 | 1200.0 | 514.3 |
| Copper (µg) | 4500.0 | 340 | 1000 | 1323.5 | 450.0 |
| Selenium (µg) | 85.5 | 20 | 90 | 427.5 | 95.0 |
| Iodine (µg) | 300.0 | 90 | 200 | 333.3 | 150.0 |
| Vitamin A (µg) | 3375.0 | 300 | 600 | 1125.0 | 562.5 |
| Vitamin D3 (µg) | 64.5 | 15 | 63 | 430.0 | 102.4 |
| Vitamin E (mg) | 71.3 | 6 | 200 | 1187.5 | 35.6 |
| Vitamin K (µg)* | 92.3 | 30 | ND | 307.5 |  |
| Ascorbic Acid (mg) | 142.5 | 15 | 400 | 950.0 | 35.6 |
| Thiamine (mg) | 1.4 | 0.5 | ND | 285.0 |  |
| Riboflavine (mg) | 4.5 | 0.5 | ND | 900.0 |  |
| Niacin (mg) | 14.3 | 6 | 10 | 237.5 | 142.5 |
| Pantothenic Acid (mg)* | 8.6 | 2 | ND | 427.5 |  |
| Vitamin B6 (mg) | 1.5 | 0.5 | 30 | 300.0 | 5.0 |
| Folic Acid (µg) | 570.0 | 150 | 300 | 380.0 | 190.0 |
| Vitamin B12 (µg) | 4.5 | 0.9 | ND | 500.0 |  |
| Biotin (µg)* | 165.0 | 8 | ND | 2062.5 |  |

RDA – Recommended Daily Allowance; AI – Adequate Intake; UL - Tolerable Upper Limit; ND – Not Determinable; *Indicates only AI (not RDA) is available for this micronutrient for this age group; ^#^UL for magnesium represent intake from a pharmacological agent only and do not include intake from food and water.

**Supplementary Table S4g:** Predicted intakes of micronutrients from RUTF for children 6-12 months of age weighing 5kg

| **Micronutrient** | **Amount Provided** | **RDA/AI** | **UL** | **%RDA/AI** | **%UL** |
| --- | --- | --- | --- | --- | --- |
| Sodium (mg)* | 288.75 | 370 | ND | 78 |  |
| Potassium (mg)* | 2049.25 | 860 | ND | 238 |  |
| Calcium (mg)* | 528.5 | 260 | 1500 | 203 | 35 |
| Phosphorus (mg)* | 600.25 | 275 | ND | 218 |  |
| Magnesium (mg)* | 140 | 75 | ND | 187 |  |
| Iron (mg) | 18.025 | 11 | 40 | 163 | 45 |
| Zinc (mg) | 20.65 | 3 | 5 | 688 | 413 |
| Copper (µg)* | 2625 | 220 | ND | 1193 |  |
| Selenium (µg)* | 49 | 20 | 60 | 245 | 82 |
| Iodine (µg)* | 171.5 | 130 | ND | 132 |  |
| Vitamin A (µg)* | 1382.5 | 500 | 600 | 277 | 230 |
| Vitamin D3 (µg)* | 24.5 | 10 | 38 | 245 | 64 |
| Vitamin E (mg)* | 32.2 | 5 | ND | 644 |  |
| Vitamin K (µg)* | 25.2 | 2.5 | ND | 1008 |  |
| Ascorbic Acid (mg)* | 80.5 | 50 | ND | 161 |  |
| Thiamine (mg)* | 0.805 | 0.3 | ND | 268 |  |
| Riboflavine (mg)* | 2.625 | 0.4 | ND | 656 |  |
| Niacin (mg)* | 8.05 | 4 | ND | 201 |  |
| Pantothenic Acid (mg)* | 4.9 | 1.8 | ND | 272 |  |
| Vitamin B6 (mg)* | 0.9625 | 0.3 | ND | 321 |  |
| Folic Acid (µg)* | 322 | 80 | ND | 403 |  |
| Vitamin B12 (µg)* | 2.625 | 0.5 | ND | 525 |  |
| Biotin (µg)* | 98 | 6 | ND | 1633 |  |

RDA – Recommended Daily Allowance; AI – Adequate Intake; UL - Tolerable Upper Limit; ND – Not Determinable; *Indicates only AI (not RDA) is available for this micronutrient for this age group

**Supplementary Table S4h:** Predicted intakes of micronutrients from RUTF for children 12-23 months of age weighing 6kg

| **Micronutrient** | **Amount Provided** | **RDA/AI** | **UL** | **%RDA/AI** | **%UL** |
| --- | --- | --- | --- | --- | --- |
| Sodium (mg)* | 346.5 | 1000 | 1500 | 35 | 23 |
| Potassium (mg)* | 2459.1 | 3000 | ND | 82 |  |
| Calcium (mg) | 634.2 | 700 | 2500 | 91 | 25 |
| Phosphorus (mg) | 720.3 | 460 | 3000 | 157 | 24 |
| Magnesium (mg) | 168 | 80 | 65^#^ | 210 | 258 |
| Iron (mg) | 21.63 | 7 | 40 | 309 | 54 |
| Zinc (mg) | 24.78 | 3 | 7 | 826 | 354 |
| Copper (µg) | 3150 | 340 | 1000 | 926 | 315 |
| Selenium (µg) | 58.8 | 20 | 90 | 294 | 65 |
| Iodine (µg) | 205.8 | 90 | 200 | 229 | 103 |
| Vitamin A (µg) | 1659 | 300 | 600 | 553 | 277 |
| Vitamin D3 (µg) | 29.4 | 15 | 63 | 196 | 47 |
| Vitamin E (mg) | 38.64 | 6 | 200 | 644 | 19 |
| Vitamin K (µg)* | 30.24 | 30 | ND | 101 |  |
| Ascorbic Acid (mg) | 96.6 | 15 | 400 | 644 | 24 |
| Thiamine (mg) | 0.966 | 1 | ND | 193 |  |
| Riboflavine (mg) | 3.15 | 1 | ND | 630 |  |
| Niacin (mg) | 9.66 | 6 | 10 | 161 | 97 |
| Pantothenic Acid (mg)* | 5.88 | 2 | ND | 294 |  |
| Vitamin B6 (mg) | 1.155 | 1 | 30 | 231 | 4 |
| Folic Acid (µg) | 386.4 | 150 | 300 | 258 | 129 |
| Vitamin B12 (µg) | 3.15 | 1 | ND | 350 |  |
| Biotin (µg)* | 117.6 | 8 | ND | 1470 |  |

RDA – Recommended Daily Allowance; AI – Adequate Intake; UL - Tolerable Upper Limit; ND – Not Determinable; *Indicates only AI (not RDA) is available for this micronutrient for this age group; ^#^UL for magnesium represent intake from a pharmacological agent only and do not include intake from food and water.

**Supplementary Table S4i:** Predicted intakes of micronutrients from RUTF for children 24-59 months of age weighing 8.5kg

| **Micronutrient** | **Amount Provided** | **RDA/AI** | **UL** | **%RDA/AI** | **%UL** |
| --- | --- | --- | --- | --- | --- |
| Sodium (mg)* | 495 | 1000 | 1500 | 50 | 33 |
| Potassium (mg)* | 3513 | 3000 | ND | 117 |  |
| Calcium (mg) | 906 | 700 | 2500 | 129 | 36 |
| Phosphorus (mg) | 1029 | 460 | 3000 | 224 | 34 |
| Magnesium (mg) | 240 | 80 | 65^#^ | 300 | 369 |
| Iron (mg) | 30.9 | 7 | 40 | 441 | 77 |
| Zinc (mg) | 35.4 | 3 | 7 | 1180 | 506 |
| Copper (µg) | 4500 | 340 | 1000 | 1324 | 450 |
| Selenium (µg) | 84 | 20 | 90 | 420 | 93 |
| Iodine (µg) | 294 | 90 | 200 | 327 | 147 |
| Vitamin A (µg) | 2370 | 300 | 600 | 790 | 395 |
| Vitamin D3 (µg) | 42 | 15 | 63 | 280 | 67 |
| Vitamin E (mg) | 55.2 | 6 | 200 | 920 | 28 |
| Vitamin K (µg)* | 43.2 | 30 | ND | 144 |  |
| Ascorbic Acid (mg) | 138 | 15 | 400 | 920 | 35 |
| Thiamine (mg) | 1.38 | 1 | ND | 276 |  |
| Riboflavine (mg) | 4.5 | 1 | ND | 900 |  |
| Niacin (mg) | 13.8 | 6 | 10 | 230 | 138 |
| Pantothenic Acid (mg)* | 8.4 | 2 | ND | 420 |  |
| Vitamin B6 (mg) | 1.65 | 1 | 30 | 330 | 6 |
| Folic Acid (µg) | 552 | 150 | 300 | 368 | 184 |
| Vitamin B12 (µg) | 4.5 | 1 | ND | 500 |  |
| Biotin (µg)* | 168 | 8 | ND | 2100 |  |

RDA – Recommended Daily Allowance; AI – Adequate Intake; UL - Tolerable Upper Limit; ND – Not Determinable; *Indicates only AI (not RDA) is available for this micronutrient for this age group; ^#^UL for magnesium represent intake from a pharmacological agent only and do not include intake from food and water.
